# The Effect of Different Surfactants and Polyelectrolytes on Nano-Vesiculation of Artificial and Cellular Membranes

**DOI:** 10.3390/molecules29194590

**Published:** 2024-09-27

**Authors:** Urška Zagorc, Darja Božič, Vesna Arrigler, Žiga Medoš, Matej Hočevar, Anna Romolo, Veronika Kralj-Iglič, Ksenija Kogej

**Affiliations:** 1 University of Ljubljana, Faculty of Chemistry and Chemical Technology, Chair for Physical Chemistry, SI-1000 Ljubljana, Slovenia; 2 University of Ljubljana, Faculty of Health Sciences, Laboratory of Clinical Biophysics, SI-1000 Ljubljana, Slovenia; 3 Institute of Metals and Technology, SI-1000 Ljubljana, Slovenia

**Keywords:** extracellular vesicles, liposomes, surfactants, polyelectrolytes, static and dynamic light scattering, microcalorimetry, drug delivery, cell-to-cell communication

## Abstract

Nano- and micro-sized vesicular and colloidal structures mediate cell–cell communication. They are important players in the physiology of plants, animals, and humans, and are a subject of increasing interest. We investigated the effect of three surfactants, N-cetylpyridinium chloride (CPC), sodium dodecyl sulfate (SDS), and Triton X-100 (TX100), and two anionic polyelectrolytes, sodium polystyrene sulfonate (NaPSS) and sodium polymethacrylate (NaPMA), on nanoliposomes. In addition, the effect of SDS and TX100 on selected biological membranes (erythrocytes and microalgae) was investigated. The liposomes were produced by extrusion and evaluated by microcalorimetry and light scattering, based on the total intensity of the scattered light (*I*_tot_), hydrodynamic radius (*R*_h_), radius of gyration (*R*_g_), shape parameter *p* (=*R*_h_/*R*_g,0_), and polydispersity index. The EPs shed from erythrocytes and microalgae *Dunaliella tertiolecta* and *Phaeodactylum tricornutum* were visualized by scanning electron microscopy (SEM) and analyzed by flow cytometry (FCM). The *R*_h_ and *I*_tot_ values in POPC liposome suspensions with added CPC, SDS, and TX100 were roughly constant up to the respective critical micelle concentrations (CMCs) of the surfactants. At higher compound concentrations, *I*_tot_ dropped towards zero, whereas *R*_h_ increased to values higher than in pure POPC suspensions (*R*_h_ ≈ 60–70 nm), indicating the disintegration of liposomes and formation of larger particles, i.e., various POPC–S aggregates. Nanoliposomes were stable upon the addition of NaPSS and NaPMA, as indicated by the constant *R*_h_ and *I*_tot_ values. The interaction of CPC, SDS, or TX100 with liposomes was exothermic, while there were no measurable heat effects with NaPSS or NaPMA. The SDS and TX100 increased the number density of EPs several-fold in erythrocyte suspensions and up to 30-fold in the conditioned media of *Dunaliella tertiolecta* at the expense of the number density of cells, which decreased to less than 5% in erythrocytes and several-fold in *Dunaliella tertiolecta*. The SDS and TX100 did not affect the number density of the microalgae *Phaeodactylum tricornutum*, while the number density of EPs was lower in the conditioned media than in the control, but increased several-fold in a concentration-dependent manner. Our results indicate that amphiphilic molecules need to be organized in nanosized particles to match the local curvature of the membrane for facilitated uptake. To pursue this hypothesis, other surfactants and biological membranes should be studied in the future for more general conclusions.

## 1. Introduction

Membrane enclosed sub-micron sized cellular fragments called extracellular vesicles (EVs) have become a subject of increasing interest as they can mediate the interaction between cells and serve as drug delivery systems [1,2,3,4,5]. The EVs are pinched off from cells at the last stage of the process of the membrane budding. They become free to travel with fluids and can be taken up by recipient cells. As they carry proteins, signaling molecules and nucleic acids or their fragments, they may become biologically active in the recipient cells. This process can be mimicked by introducing synthetic nanoparticles (e.g., liposomes, inorganic nanoparticles, and different combinations—hybridosomes) which enclose healing substances within artificial membranes. This process is the principle of drug delivery to cells. The EVs, liposomes, and hybridosomes are subject to the same physical laws and can mediate interactions between all living systems and the environment. They present a fundamental level of the One Health concept. Understanding and mastering the properties of these structures and their interactions with the membrane is, therefore, highly warranted in medicine, agriculture, food industry, and environmental sciences.

The biological membrane is composed of a hydrophobic interior and two hydrophilic interfaces (in contact with the outer and the inner cellular solution); therefore, amphiphilic molecules in such systems act as surfactants, which may have an important impact on the membrane. Liposomes are nano-sized vesicles in which lipid membrane encloses structureless interior that may be loaded for delivery to cells. Previous studies have shown notable effects of surfactants on the shape and integrity of erythrocytes [6,7] and artificial membranes [8,9].

In this work, we focused on selected surfactants (N-cetylpyridinium chloride [CPC], sodium dodecyl sulfate [SDS], and Triton X-100 [TX100]) and anionic polyelectrolytes, sodium polystyrene sulfonate (NaPSS), and sodium polymethacrylate (NaPMA). The surfactants were either ionic (CPC and SDS) or nonionic (TX100). The cationic surfactant, CPC, is an amphiphilic compound that has been widely used in personal care products as it was found to have antiseptic properties. It was suggested that the mechanism of destruction of micro-organisms is the disruption of the lipid bilayer—the base of the membrane [10,11,12,13,14]. The anionic surfactant, SDS, was found to be involved with certain peptides in the formation of amphiphilic ordered structures (α-helixes and β-sheets) and the induction of helical folding in some non-helical proteins [6]. The nonionic surfactant, TX100, has been frequently reported to lyse cells to extract proteins and organelles or to permeabilize the living cell membrane for transfection [15]. As it interacts with the cellular membrane, its larger quantity causes the destruction of the compactness and integrity of the lipid membrane [16,17]. Amphiphilic polymethacrylate copolymers were designed to modify lipid bilayers [18] by their fragmentation. It was observed that they induced the formation of lipid nanodiscs [19]. The NaPSS is a non-surface-active polyelectrolyte. It has a hydrophobic phenyl moiety and hydrophilic sulfonate group and can interact with cationic surfactants both hydrophobically and electrostatically [20,21,22].

**Figure 1:**
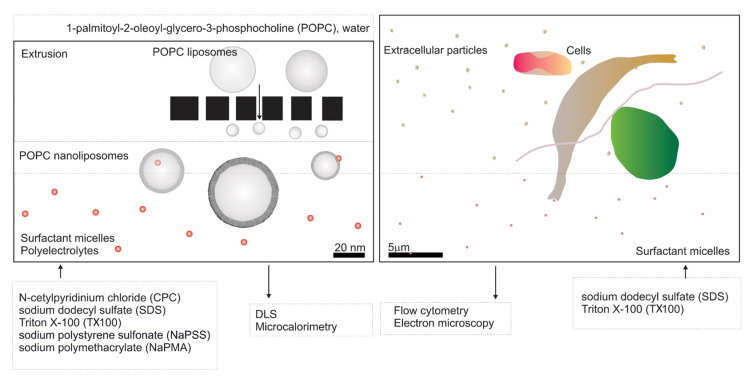
Scheme of the study. (**Left**) study of POPC liposome membrane stability; (**right**) study of cellular membrane vesiculation.

We have included nanoliposomes in the study of the effect of the above listed surfactants and polyelectrolytes on membranes. The general scheme of our study is presented in Figure 1. Dynamic/static light scattering (LS) [23] and microcalorimetry [24,25] (Figure 1, left panel) have proven to be relevant methods to characterize the samples and interactions within various colloidal systems. We have followed the effect of these compounds on the size and stability of nanoliposomes prepared from 1-palmitoyl-2-oleoyl-sn-glycero-3-phosphocholine lipid (POPC) by extrusion. In addition, the interaction of the compounds with POPC liposomes was studied by microcalorimetry. To the best of our knowledge, microcalorimetry, in combination with LS, was used for the first time in stability studies of POPC liposomes in surfactant (S)/polyelectrolyte (PE) mixed systems. Of great interest is the study of the mechanisms of membrane budding and vesiculation [26,27,28,29,30,31]. We followed the effects of SDS and TX100 on vesiculation of cell membranes ex vivo (in erythrocytes) and in vitro (in microalgae) by electron microscopy and flow cytometry (Figure 1, right panel). Micro-organisms in the environment are exposed to various amphiphilic substances. The interaction of their membranes with surfactants can result in the leakage and release of cellular contents into the extracellular environment [32,33]. Once inside the cell, the surfactants can disrupt protein arrangements and enzyme activity of microalgae [34]. It is, therefore, very important to understand the effects of surfactants on microalgae.

## 2. Results and Discussion

### 2.1. CMC of Surfactants

The CMC of CPC in the TRIS buffer was 0.6 mM (600 µM) and the CMC of SDS in the TRIS buffer was 7.55 mM (7550 µM). Both values were determined by conductivity measurements (c.f. Figure S1 in Supporting Material [SM]). These values are slightly lower than those in water at the same temperature (0.63 mM for CPC [21] and 8.2 mM for SDS [35], both at 25 °C). Such a result was expected given the low buffer concentration, i.e., low ionic strength of the solvent. The CMC value of TX100 in water at 25 °C is between 0.22 and 0.24 mM (220–240 µM [36]), which is the lowest of all the surfactants used in this study, and was taken as independent of ionic strength.

### 2.2. Size and Shape of Pure POPC Liposomes

Static (SLS) and dynamic light scattering (DLS) methods were used for the size and shape characterization of pure/intact POPC vesicles (Figure S2). Hydrodynamic radius, *R*_h_ (a DLS parameter); radius of gyration, *R*_g_ (an SLS parameter); and the shape factor, *ρ* = *R*_g_*/R*_h_(0) (where *R*_h_(0) is the *R*_h_ value obtained by extrapolation of *R*_h_ to *q* = 0 (c.f. Figure S2B)) of pure POPC liposome suspensions in 5 mM TRIS buffer at 25 °C are presented in Table S1 and the corresponding plots are shown in Figure S2. For the case shown in Figure S2, the obtained values were as follows: *R*_h_(0) = 78.5 nm, *R*_g_ = 76.4 nm, and *ρ* = 0.97. These values were expected for spherical particle topology characteristic for liposomes with mass concentrated on the rim and much lower internal density (the theoretical *ρ*-value of a hollow spherical particle is *ρ* = 1). There was no sign of aggregation in pure POPC suspensions (c.f. Figure S2A and comments in SM). Other values of *ρ* were in the range between 0.96 and 1.00 (c.f. Table S1), agreeing with the spherical shape and known mass distribution for liposomes.

### 2.3. Effect of Surfactants and Polyelectrolytes on Liposome Size, Shape, and Stability

**Figure 2:**
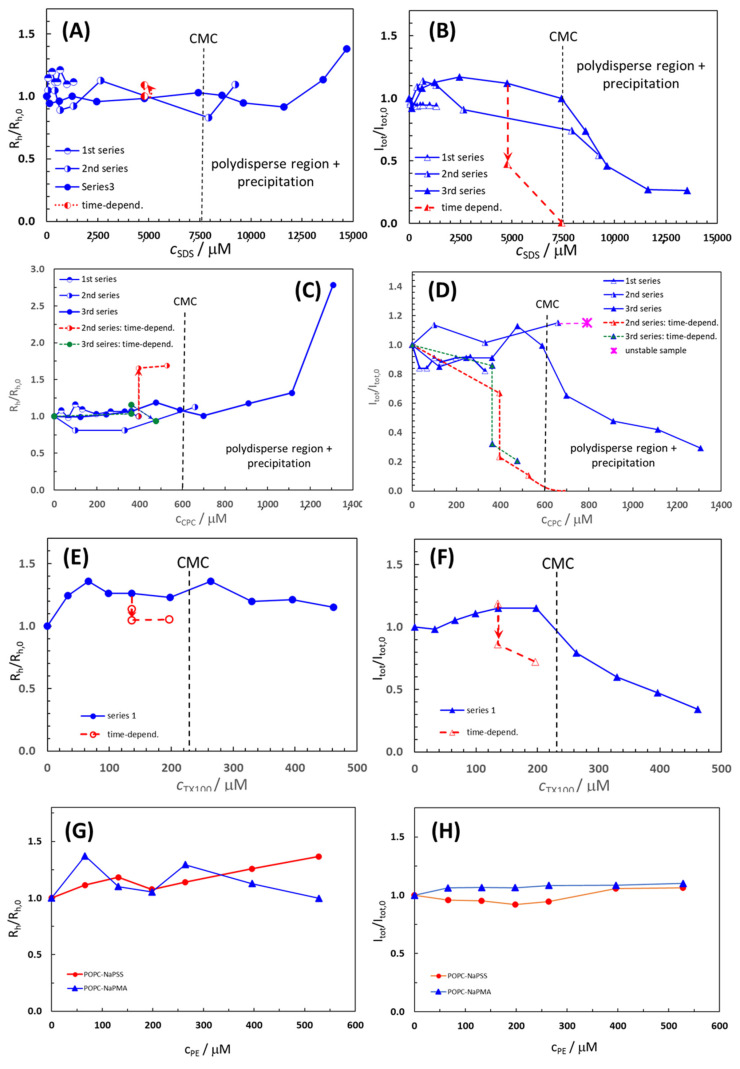
The average *R*_h_*/R*_h,0_ values of the POPC liposome population and the total intensity of light scattered from POPC suspensions at an angle of 90°, presented as the ratio *I*_tot_/*I*_tot,0_, as functions of the added compound concentration; (**A**,**B**) SDS; (**C**,**D**) CPC; (**E**,**F**) TX100, (**G**) NaPSS; and (**H**) NaPMA. The *R*_h,0_ is the hydrodynamic radius, and *I*_tot,0_ is the total intensity of scattered light in pure POPC liposome suspensions. Time-dependence data (obtained after 24 h) are depicted by the dashed red lines in Panels (**A**–**F**). The nominal concentration of POPC in suspensions was 132 µM. The black vertical dashed lines in Panels (**A**–**F**) indicate the CMC values of surfactants in 5 mM TRIS buffer at 25 °C.

The average *R*_h_*/R*_h_(0) values of the POPC liposome population and the total intensity of light scattered from POPC suspensions at an angle of 90°, presented as the ratio *I*_tot_/*I*_tot,0_, in the absence and presence of added surfactants and polyelectrolytes are shown in Figure 2. Here, *R*_h,0_ is the hydrodynamic radius, and *I*_tot,0_ is the total intensity of scattered light of pure POPC liposome suspensions. Raw data on *R*_h_*/R*_h,0_ and *I*_tot_/*I*_tot,0_ are collected in Tables S2–S6 for all the systems studied.

For polydisperse samples containing more than one population of particles, the *R*_h_ value of the population with the highest contribution to *I*_tot_ is plotted in Figure 2, which mostly applies to extruded POPC liposomes with the initial *R*_h,0_ around 60–70 nm. For CPC and SDS, the measurements were performed in three series by changing the width of the surfactant concentration, whereas for TX100 (and for both PEs) only one series was carried out. In at least one series of measurements, the concentration range of the surfactant was such that its CMC (marked by vertical lines in Figure 2) was sufficiently exceeded.

The *R*_h_*/R*_h,0_ and *I*_tot_/*I*_tot,0_ values in POPC liposome suspensions with added CPC, SDS, and TX100 were roughly constant at *R*_h_*/R*_h,0_ (*I*_tot_/*I*_tot,0_) = 1.0 ± 0.2 up to the respective CMC or even above it. At higher compound concentrations, *I*_tot_/*I*_tot,0_ dropped towards zero, whereas *R*_h_*/R*_h,0_ started to increase to values higher than 1, indicating the disintegration of liposomes and formation of larger particles, i.e., various POPC–S aggregates. The least expressed was the decrease in *I*_tot_/*I*_tot,0_ (accompanied by a decrease in *R*_h_*/R*_h,0_ in this case) above the CMC for TX100, indicating that liposomes are the most resistant to the addition of this nonionic surfactant.

The initial constancy of *R*_h_*/R*_h,0_ and *I*_tot_/*I*_tot,0_ in the presence of CPC, SDS, and TX100 suggests that during the time of the measurements (total time for one series was around 100 min) the surfactant has not yet begun to disintegrate liposomes or, to a greater extent, incorporate into them. The incorporation becomes significant only when the compound’s CMC is strongly exceeded or after longer times following compound addition. Time stability was tested for suspensions with compound concentrations below the CMC (c.f. the red points and lines in Figure 2). In these conditions, POPC suspensions with added compounds were free from visible precipitation. After letting the suspensions stand for 1 day, *I*_tot_/*I*_tot,0_ dropped steeply and *R*_h_*/R*_h,0_ increased (SDS and CPC; Figure 2A–D) or decreased (TX100; Figure 2E). In the POPC–SDS sample with *c*_SDS_ = 4.77 mM, *I*_tot,0_ significantly decreased while *R*_h_ slightly increased (Figure 2A,B), whereas with further SDS addition to *c*_SDS_ = 7.42 mM, *I*_tot_ dropped to 0 and *R*_h_ could not be measured due to poor quality of the correlation function, indicating the precipitation of the POPC–SDS complex from the solution. The *R*_h_ distributions for *c*_CPC_ > 700 µM (c.f. the third series) were multimodal, displaying several peaks. In addition to small particles (*R*_h_ < 10 nm), very large ones (*R*_h_ > 1 mm) were also formed, which led to visible and extensive precipitation in the cuvette and subsequent phase separation of the mixed POPC–CPC aggregates from the solution.

In the POPC–TX100 system, *R*_h_*/R*_h,0_ increased with the first two additions of TX100 (up to *R*_h_*/R*_h,0_ = 1.36), but remained approximately constant afterward. The initial increase can be related to the incorporation of TX100, with a rather large head group (c.f. discussion below) into the POPC bilayer and a simultaneous increase in the liposome diameter. Above the CMC, smaller particles (*R*_h_ < 10 nm) were formed in the POPC–TX100 suspension, which could be lipid–TX100 micelle-like clusters, whereas formation of larger POPC–TX100 aggregates with *R*_h_ of a few 100 nm (*D*_h_ ≈ 1 µm) was detected only for the last two points in Figure 2E,F (*c*_TX100_ = 400 µM and 460 µM, respectively) and when following time dependence (for *c*_TX100_ = 136 µM and 196 µM, respectively). In the latter case, *R*_h_ slightly dropped whereas *I*_tot_ decreased, but dropped much less than in samples with CPC or SDS. The TX100 favored the formation of smaller mixed micelles with POPC.

To summarize, the observed increase in *R*_h_*/R*_h,0_ and decrease in *I*_tot_/*I*_tot,0_ suggest that SDS, CPC, and TX100 molecules were notably incorporated into the lipid bilayer of the liposomes when they were apt to form micelles, i.e., above their respective CMCs. In the initial stage, when the amount of the added surfactant was low (below the CMC), this did not affect the liposomes, at least not immediately, and liposome size remained approximately constant. The breakdown of the POPC liposomes took place even below the CMC, but this process was relatively slow. However, as the concentration of the surfactant increased and approached the CMC, the liposome size increased (in particular with added CPC) and the process led to the degradation of the liposomes. The lipid and the surfactant favored forming mixed micelles that were smaller than liposomes (*R*_h_ ≈ 10–30 nm), but also large aggregates (*R*_h_ ≈ several 100 nm or even above 1 μm) that ultimately precipitated from suspensions. Both the liposomes and the mixed micelles coexisted in the solution for some time. Based on this, we conclude that the surfactant must be in the micellar form for the uptake by the liposomes. At the beginning of this integration, when liposome curvature is not yet strongly affected by the embedded surfactant clusters, the bilayers are rather stable. However, when the curvature grows too much, the surfactant leads to the disintegration of the liposomes and reorganization of the amphiphilic material into other associated structures. The process seems to depend on the size of the interacting material, and clusters of surfactant and lipid, which should be in the nanometer size range, meaning that individual surfactant molecules are too small. Micelles, having appropriate curvature and size, can be viewed as a kind of “nano-snacks” that are easily eaten by the liposomes. Finally, a comparison of the LS data for the non-ionic surfactant TX100 and the two ionic surfactants suggests that the effect of TX100 on the stability of POPC liposomes is the mildest of the three surfactants. This distinction could be associated with the structure of TX100, having a polar “tail-like” head group much larger in comparison to the nonpolar tail. The “tail-like” polar head group protrudes out of the liposome outer surface, which contributes to a more pronounced increase in liposome size in this case.

The *R*_h_ of POPC liposomes increased with the addition of PE (from 61 nm in pure POPC suspension to around 83 nm in a suspension with *c*_NaPSS_ = 528 µM or *c*_NaPMA_ = 66 µM), whereas *I*_tot_ remained constant, indicating that there is no extensive breakdown of the liposomes by PEs. Smaller particles (*R*_h_ ≈ 10–30 nm) were observed at the three highest PE concentrations but we detected no large particles and no precipitation. We suggest that the increase in *R*_h_, in this case, is a consequence of the adsorption of PE chains on the surface of the liposome, which seems the most likely situation from the point of view of the negative charge of the highly water-soluble PE chains. Smaller particles may be individual PE chains or compact smaller complexes composed of PE chains and lipid molecules or their smaller clusters. Such complexation could be a result of the attractive electrostatic interactions between the anionic PEs and lipid molecules carrying a positive charge on the nitrogen atom in the polar head group close to the surface of the POPC bilayer [37]. We conclude that the anionic polyelectrolytes, NaPSS and NaPMA, did not significantly affect POPC liposome size and stability within the system parameters studied.

### 2.4. Effect of Temperature on Size and Stability of Liposomes with Added Surfactants and Polyelectrolytes

**Figure 3:**
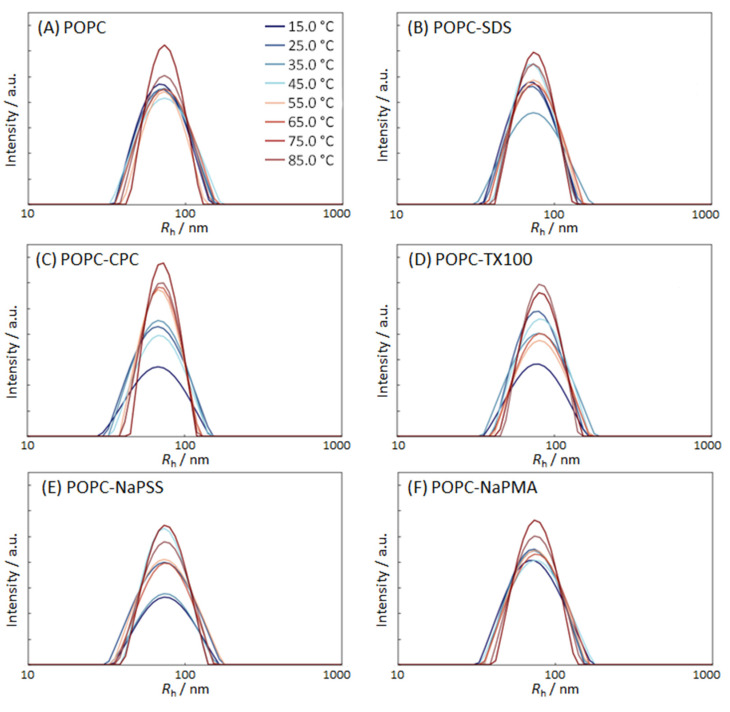
Distribution of the hydrodynamic radii *R*_h_ of POPC liposomes in 5 mM TRIS buffer suspensions (pH = 8) at different temperatures (indicated in the plots); (**A**) pure POPC suspensions; (**B**) POPC suspensions with added SDS; (**C**) POPC suspensions with added CPC; (**D**) POPC suspensions with added TX100; (**E**) POPC suspensions with added NaPSS; and (**F**) POPC suspensions with added NaPMA. The nominal POPC concentration was *c*_POPC_ = 132 µM and the nominal compound:POPC molar ratio was 1:1. Temperature legend is the same for all Figures.

The effect of temperature was followed for POPC suspensions with the equimolar (1:1) ratio between the added compound and POPC; this was always below the CMC of the surfactant. The DLS experiments at 25 °C indicated stability of the samples in the time interval of a few hours (see above). The *R*_h_ distribution curves of POPC liposome suspensions without and with added Ss or PEs at different temperatures and *c*_POPC_ = 132 µM are shown in Figure 3 and the respective raw data are given in Table S6.

The POPC liposome suspensions without additives were found to be extremely stable with respect to heating. The *R*_h_ distribution curves in Figure 3A exhibited a single broad peak corresponding to one population of particles. The position of the peak was almost independent of temperature while the width of the distribution decreased with increasing temperature. The narrowest and the highest distribution was observed at the highest temperature (85 °C). Also, in samples with added compounds at concentrations below the CMC, the average particle size did not change considerably, and the liposome size distributions became narrower with increasing temperature. By comparing *R*_h_ distributions of pure POPC liposomes with those in the presence of added compounds, we see that the latter are generally broader at low temperatures and narrower at high temperatures. It could be speculated that smaller mixed POPC–S aggregates in the samples are less stable than larger liposomes. They are, therefore, more affected by heating. Increased kinetic energy at higher temperatures enables the rearrangement of species into energetically favored complexes, and so the mixed micelles disappear (are “eaten” by the liposomes). How lipid and surfactant molecules are spread among the various associated species in solution and their relative stability is also important. The overall molar ratio of the surfactant to the lipid in the suspension was 1:1, but the distribution among various structures is poorly understood. At lower temperatures, POPC suspensions with added compounds were more polydisperse with respect to size, and at higher temperatures, they were more homogeneous. From the data in Table S6, we also see that the addition of CPC and SDS to a POPC suspension led to some compaction of the liposomes (average *R*_h_ was lower in comparison with the population of pure POPC) whereas the addition of TX100 increased liposome size. As explained above, this result could be attributed to the large size of the polar head group of TX100 that protrudes out of the liposome surface.

### 2.5. Calorimetric Measurements of POPC/Surfactant and POPC/Polyelectrolyte Colloids

Before reaching the surfactant’s CMC in the titration cell, the injected micelles break apart into individual molecules, so called unimers. Most of the measured heat (at constant pressure, i.e., Δ*H*) is assumed to be associated with the corresponding process of (de)micellization. However, at 25 °C, the enthalpy of (de)micellization for all the studied surfactants was very small (close to 0). Therefore, the measurements were conducted at 15 °C, where the heat effect of (de)micellization was found to be stronger [25]. The (de)micellization heat is combined with other contributions, namely, the heat of ion, micelle, liposome dilution (the dilution enthalpies were measured separately and subtracted from the total heat effect as described in the Materials and Methods), the heat-related to structural changes of either micelles or liposomes, and the heat effect related to the interaction of the added compound with liposomes. Due to the complex nature of all these interactions, the results are discussed qualitatively without an attempt to evaluate the heat effects of separate processes.

**Figure 4:**
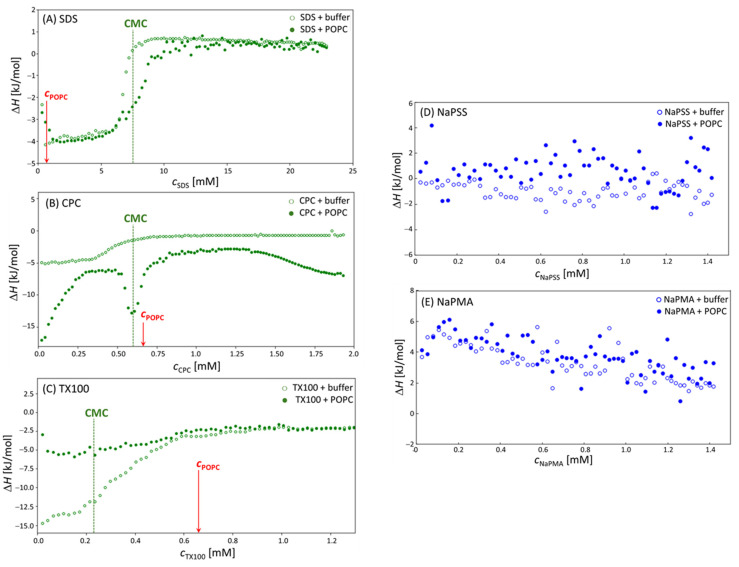
Enthalpograms obtained by titrating (**A**) SDS; (**B**) CPC; (**C**) TX100; (**D**) NaPSS; and (**E**) NaPMA solution (empty circles) into 0.66 mM POPC suspension (filled circles) in 5 mM TRIS buffer. The vertical dashed lines indicate the surfactant’s CMC and the red arrows indicate the concentration of POPC (*c*_POPC_). All data were collected on the VP–ITC calorimeter at 15 °C.

The obtained enthalpograms for experiments involving stock solutions of surfactants titrated into the solution of POPC liposomes at 15 °C on VP–ITC are shown in Figure 4A–E). Each Panel in Figure 4 contains the following two experiments: the (de)micellization of the surfactant in 5 mM TRIS buffer (open symbols) and the same surfactant stock solution titrated into the 0.66 mM POPC liposome suspension. For comparison, measurements at 25 °C, obtained on Nano ITC, are shown in Figures S3–S5. Due to larger heat effects at 15 °C, the discussion is focused on the results at 15 °C.

The curves in the case of all three surfactants in the absence of POPC liposomes (open symbols) are typical for the (de)micellization of surfactant with the pre-CMC nearly linear at the part where surfactant micelles fully break apart in the POPC-free solvent, the post-CMC part where nearly constant heat is observed due to the dilution of the micelles, and the inflection point in-between where surfactant micelles and unimers co-exist [25]. All three surfactants have negative (exothermic) (de)micellization heat effects with TX100 displaying the largest ones. The plateau Δ*H* values are around −13 kJ/mol for TX100 and between −4 and −5 kJ/mol for SDS and CPC.

In the presence of POPC liposomes, the curves change. These changes are related to the relative position of the surfactant’s CMC (indicated by the dashed vertical lines) and the value of *c*_POPC_ (indicated by red arrows). In the CPC case, CMC and *c*_POPC_ are close; in the SDS case, *c*_POPC_ is much lower than the CMC and with TX100 it is higher. The inflection point in the calorimetric curves in mixed systems is always observed in the vicinity of the CMC, whereas the ratio CMC/*c*_POPC_ determines the width of the plateau. Thus, the plateau (or better, the maximum) in Δ*H* is rather narrow when CMC *c*_POPC_ ≈ 1 (the CPC case) and much broader when it is rather different, either low (TX100:CMC/*c*_POPC_ ≈ 0.35) or high (SDS:CMC/*c*_POPC_ ≈ 11).

In the case of SDS, *c*_POPC_ is considerably lower than the CMC (SDS is in large excess with respect to POPC); therefore, a large part of the initial incorporation of SDS into POPC bilayers (the pre-CMC interaction) is not covered. In the pre-CMC region, SDS forms almost pure SDS micelles and, thus, presents very similar heat effects of (de)micellization as in pure SDS solutions, because it is in a much larger excess with respect to POPC (CMC/*c*_POPC_ ≈ 11) as compared to CPC (CMC/*c*_POPC_ ≈ 0.9). Due to the incorporation of SDS unimers into liposomes in the pre-CMC region, it is expected that free SDS concentration in solution would decrease. This shifts the inflection point (the CMC) to higher total surfactant concentrations in the presence of POPC. In the post-CMC region, both curves almost overlap again because the amount of the lipid in SDS–POPC solutions is below 10 mol%.

In samples with CPC, the maximum in Δ*H* separates the pre-CMC and post-CMC regions. When CPC micelles are titrated into POPC liposomes, they are immediately incorporated into POPC bilayers. This process led to increased local and global curvature of the membrane, which is usually exhibited as the budding/sprouting effect. Eventually, various associated structures, which contain both the surfactant (CPC) and the lipid (POPC) in comparable amounts, were formed. Contributions of these processes seem to be strongly exothermic. At the CPC:POPC ratio around 1:1, the total heat effect had a maximum. The first process (association of CPC with POPC) reduces the amount of free surfactant in solution and “shifts” the effective CMC for the formation of mixed CPC–POPC micelles (c.f. the second inflection point) to a higher overall *c*_CPC_, where a larger than expected heat effect is observed. Most likely this effect is due to the competition of CPC association with the liposomes and the formation of its own micelles or mixed CPC–POPC micelles/aggregates. At approximately 2-times the CMC value of CPC, the measured heat effect begins to increase in absolute value indicating further structural changes of these aggregates.

The TX100 is a non-ionic surfactant with a diverse composition due to its polymerization origin and a rather low CMC. Besides, the POPC concentration is almost 3-times higher than the surfactant’s CMC in this case. The curve for pure TX100 resembles the one for SDS; however, the (de)micellization enthalpy is much larger for TX100 (more than 3-times) than for SDS and the inflection region (coexistence of unimers and micelles) is broader. In the presence of POPC, the absolute value of (de)micellization enthalpies decreases considerably (enthalpy increases from −15 kJ/mol [pure TX100] to −5 kJ/mol [mixed POPC–TX100 solutions]). The aggregates/mixed micelles that form between TX100 and POPC are probably rich in POPC. These particles could be rather different (in shape and size) from pure TX100 micelles; therefore, the Δ*H* value is expected to be different. The lower absolute Δ*H* values indicate a less favorable packing of POPC and TX100 molecules into mixed aggregates, which again contributed to a large polar headgroup of TX100.

Figure 4D,E show enthalpograms measured in the case of NaPSS and NaPMA. The data for pure PE samples and POPC–PE mixed systems overlap. The Δ*H* values are close to 0 in the NaPSS case, but slightly positive with NaPMA. These calorimetry results suggest no significant structural changes in POPC–PE suspensions or interaction between POPC liposomes and both PEs. Again, a more detailed discussion is hampered because Δ*H* values contain contributions from various processes. It may be that all these contributions are rather low and/or that they cancel out. However, the calorimetric result on no or weak interaction agrees with the DLS measurements. The exhibited mild interaction of linear polyelectrolytes with liposomes is exploited for the protection of bilayers against degradation. To sum up, heating of the samples caused the pure POPC and the combined systems to become more homogeneous in size which indicates that the system is dynamic and that its configuration is based on physical laws enabling an accelerated approach to the equilibrium in systems with higher kinetic energy of the constituents. The latter was supported by microcalorimetry data.

To conclude the first part (POPC–S(PE) mixed systems) of our report, we add a comparison of the above results with related works on interactions of various lipid systems with surfactants and polyelectrolytes in the literature. In fact, a direct comparison is quite difficult because the employed lipids, surfactants, and polyelectrolytes, and the experimental approaches, differ from our study [38,39,40]. For example, physical stability of liposomes prepared from egg–PC and α-tocopherol was examined by the zeta potential and isothermal titration calorimetry (ITC) measurements at various amounts of added non-ionic polysorbate surfactants (Tween 20 and Tween 80) [38]. Those experimental results demonstrated that adding the Tween surfactants increased the attractive interaction potential between the liposomes, but did not change their zeta potential. In another study, interactions between nonionic Triton X surfactants with various sizes of the polar head group and cholesterol-containing phosphatidylcholine liposomes were investigated by measuring an empirical liposome stability ratio [39]. Authors showed that the effectiveness of Triton X surfactants in solubilizing vesicles increases with decreasing polyethylene glycol chain length of surfactants. The Triton X surfactant with the lowest number of ethylene glycol units per molecule (i.e., TX100, the same as in our study) exhibited the highest solubilization power. As far as the polyelectrolytes are concerned, interactions between various hydrophilic polymers (anionic sodium alginate and carboxymethyl cellulose sodium salt, and a nonionic poly [vinyl alcohol]) and liposomes composed of hydrogenated soybean lecithin were investigated by similar methods as in our study, i.e., by means of ITC and DLS measurements [40]. In agreement with our results, the authors found that adsorption of the polymers onto the liposome surfaces seemed to be very small, but still contributed to stabilization of the liposomes. It should be stressed, however, that the composition and purity of the lipids in these studies are often unknown [38,40], which makes quantitative comparison very difficult.

### 2.6. Effect of SDS and TX100 on Vesiculation of Membranes of Erythrocytes and Microalgae

**Figure 5:**
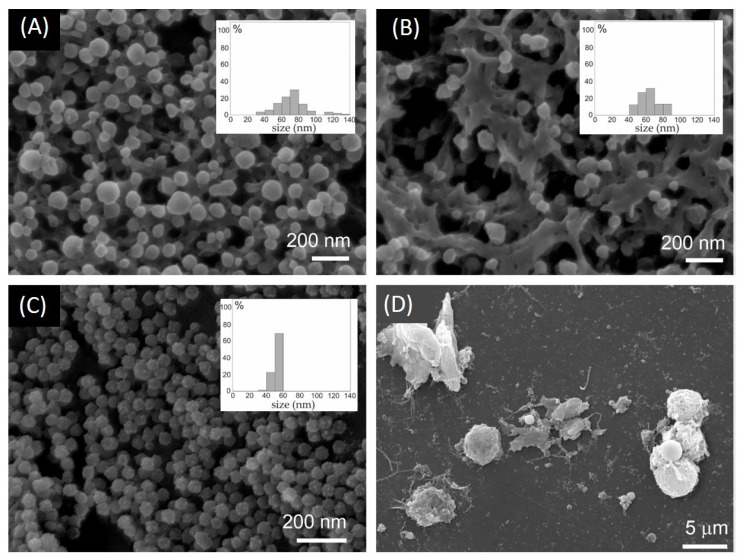
(**A**) EVs isolated from aged erythrocyte suspension stimulated by 50 mM SDS; (**B**) EVs isolated from aged erythrocyte suspension stimulated by 50 mM SDS and incubated at 80 °C; (**C**) EVs in the *Phaeodactylum tricornutum* culture; (**D**) *Dunaliella tertiolecta* culture stimulated by TX100. Insets in (**A**–**C**) show histograms of particle sizes.

Cellular membranes are prone to form nanostructures such as tunneling nanotubes and non-sized buds. It is, therefore, of interest to observe the performance of the compounds tested on POPC liposomes in complex living systems, i.e., erythrocytes and the following two types of microalgae: *Phaeodactylum tricornutum* and *Dunaliella tertiolecta*. We were particularly interested in the nanovesiculation process which may be different in different cells. Figure 5 shows EPs in isolates from aged erythrocytes, and EPs in both microalgae cultures. Erythrocyte isolates (Figure 5A,B) were rich in EVs having smooth globular shapes. The sample treated with high temperature (Figure 5B)) shows that many EVs were preserved. The EPs from the *Phaeodactylum tricornutum* culture were homogeneous in size and shape and unlike erythrocyte vesicles had a rough surface (Figure 5C). Figure 5D shows *Dunaliella tertiolecta* culture treated with TX100. The cells are decaying and many fragments that were heterogeneous in size and of irregular shape can be seen.

**Figure 6:**
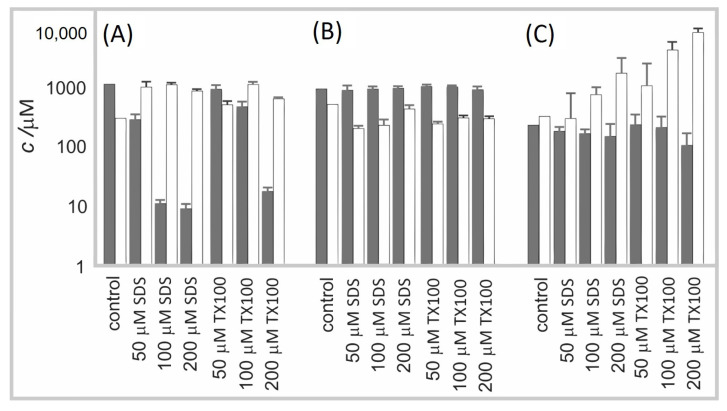
The FCM analysis of the effect of surfactants SDS and TX100 on (**A**) erythrocyte suspension; (**B**) culture of microalgae *Phaeodactylum tricornutum*; and (**C**) culture of microalgae *Dunaliella tertiolecta*. The number density of cells is shown by gray bars and the number density of EPs by white bars.

Figure 6 shows the effect of surfactants SDS and TX100 on erythrocytes and the following two types of microalgae: *Phaeodactylum tricornutum* and *Dunaliella tertiolecta*. In the untreated erythrocyte suspension, the number density of erythrocytes was the highest and the number density of EPs was the smallest. Treatment of the suspension with a low concentration of surfactant caused a moderate decrease in the number density of erythrocytes and a moderate increase in the number density of EPs. At higher concentrations of surfactants, the number density of erythrocytes significantly decreased. The SDS was detrimental already at lower concentrations than TX100. The number density of microalgae *Phaeodactylum tricornutum* in the conditioned media was insensitive to the surfactants. The number density of EPs was, however, somewhat lower than in the control. A slight concentration-dependent trend can be observed (Figure 6B)). In the *Dunaliella tertiolecta* culture, the treatment with surfactants diminished the number density of cells in a concentration-dependent way. Concomitantly, the number density of EPs considerably increased, and the effect strongly depended on the concentration of the surfactant. Here, in turn, TX100 had a greater effect than SDS.

It can be concluded that SDS and TX100 had a concentration–dependent effect on erythrocyte and *Dunaliella tertiolecta* integrity and vesiculation/fragmentation. We did not notice changes in the number density of cells in *Phaeodactylum tricornutum*. However, microalgae from the *Dunaliella* genus do not have a rigid polysaccharide cell wall that imposes the cell shape and are, therefore, more prone to adjust, to some extent, their volume and shape in response to changes in the environment [41] than *Phaeodactylum tricornutum*, which has a rigid cell wall composed mostly of sulfated glucuronomannan, polysaccharides, proteins, long-chain polyamines, and lipids [42,43]. In regard to physiological criteria to define cell viability of phytoplankton cells [44,45], disabling the trans-membrane transport and the loss of physical integrity of the plasma membrane were suggested to be essential to distinguish dead from live cells [46]. The surfactants could be affecting the cell viability of *Phaeodactylum tricornutum*, which was not detected in FSC/SSC FCM scatter diagrams. Furthermore, the detection of the number density of EPs by FCM was limited to large EPs as the instrument used has a threshold of about 400 nm for FSC/SSC assessment. While in *Dunaliella tertiolecta* the fragmentation of cells resulted in a population heterogeneous in size and shape of EPs, in erythrocytes and *Phaeodactylum tricornutum*, the population of EPs is expected to be more homogeneous and the average size smaller (Figure 5); complementary techniques should be used to detect EPs of that size [35].

Of great interest is the study of mechanisms of cell membrane budding and vesiculation [26], which may be different in different types of cells. Exo-vesiculation of the erythrocyte membrane is preceded by the shape transformation of the erythrocyte into an echinocyte, formation of spicules, detachment of membrane skeleton, formation of the buds at the top of the spicules, narrowing of the neck connecting the bud and the mother membrane, and, finally, detachment of the bud from the membrane to create free vesicles [26]. In microalgae, the mechanisms are currently obscure, however, harvesting and characterization of nano-sized extracellular particles (EPs) have been accomplished in some species [27,28,29]. Enormous quantities of surfactants are used daily for industrial and household purposes and are released into sewage systems to be degraded or directly into surface waters [45]. Micro-organisms in the environment are exposed to these remnants and the interaction of their membrane with surfactants can result in the leakage and release of cellular contents into the extracellular environment [32,33]. Once inside the cell, the surfactants can disrupt protein arrangements and enzyme activity of microalgae, which can affect the organization of the thylakoids and interfere with chlorophyll synthesis [34]. It is of utmost importance to better understand the effects of surfactants on microalgae.

## 3. Materials and Methods

### 3.1. Materials

#### 3.1.1. Chemicals

The 1-Palmitoyl-2-oleoyl-phosphatidylcholine (POPC, *M* = 760.076 g/mol) was purchased from Avanti Polar Lipids Inc. (Alabaster, AL, USA) and used as received. The N-cetylpyridinium chloride (CPC, *M* = 760.076 g/mol) and sodium dodecyl sulfate (SDS, *M* = 288.372 g/mol) were purchased from Merck (Rahway, NJ, USA) and thoroughly purified by repeated recrystallization from acetone and dried under vacuum at 50 °C. The Triton X100 (TX100; *M* = 647 g/mol) was purchased from Sigma Aldrich (St. Louis, MO, USA) and was used as received. The sodium polystyrene sulfonate (NaPSS; weight average molecular mass *M*_w_ = 149 000 g/mol and polydispersity index PDI = 1.2) was purchased from Honeywell Research Chemicals (Fluka, Morris Plains, NJ, USA) and sodium polymethacrylate (NaPMA; *M*_w_ = 205 000 g/mol and PDI = 1.3) was purchased from Polymer Source Inc. (Montreal, QC, Canada). Both polyelectrolytes were used as received. All other salts and reagents were of analytical grade and were used as supplied. All solutions were prepared in triply distilled water, designated as dH_2_O.

#### 3.1.2. Preparation of TRIS Buffer

The 5 mM TRIS buffer (pH = 8) was prepared by weighing 151.5 mg TRIS into a 250 mL volumetric flask and adding distilled water. After dissolving the buffer components, the pH was measured, 1 M HCl was added to adjust the value to pH = 8, and the flask was filled to the mark with dH_2_O. Before use, the buffer solution was filtered through a 1.2 mm pore size filter and stored at 4 °C.

#### 3.1.3. Preparation of the Stock POPC Liposome Suspension

The POPC liposomes were prepared by the freeze–thaw extrusion method. A round bottom flask was filled with 1.3 mL of 10 mg/mL POPC dissolved in a chloroform/methanol (2:1, *v*/*v*) mixture. By rotating the flask, the chloroform evaporated, and a lipid film spread evenly on the flask wall. The remaining organic solvent was removed by drying the film at a reduced pressure at 40 °C overnight. The dry lipid film was hydrated with 1.3 mL of the TRIS buffer for 24 h and then shaken ultrasonically. The nominal concentration of POPC in the suspension before extrusion was *c*_POPC_ = 13.16 mM. One mL of this suspension was passed 19 times through two polycarbonate membranes with a pore size of 100 nm mounted in an Avanti Polar Lipids extruder (USA). The extrusion was carried out at room temperature. Afterward, the sample was stored in a cryogenic vessel. The concentration of POPC after extrusion was not determined. The value *c_P_*_OPC_ = 13.16 mM was taken as the nominal POPC concentration.

#### 3.1.4. Preparation of Surfactant (S) and Polyelectrolyte (PE) Solutions

The solutions of surfactants and polyelectrolytes were prepared by weighing the corresponding amounts of compounds and adding 5 mM TRIS buffer so that the final concentration of S or PE was 10 mmol/L (in the case of PE, mol refers to the repeating units of PE and not to the whole chain). Since SDS has a higher critical micelle concentration (CMC) than CPC or TX100, the concentration of the SDS solution was 10-times higher, i.e., 100 mmol/L. Other specific details regarding solution preparation are given in the method descriptions below. The CMC values of ionic surfactant (CPC and SDS) in the 5 mM TRIS buffer were determined by conductometry (see below).

#### 3.1.5. Preparation of POPC Suspensions with Added S or PE for Light Scattering Measurements

The DLS measurements were performed at a constant POPC concentration (nominal value *c*_POPC_ = 132 mM) obtained by a 100-fold dilution of the stock POPC suspension with 5 mM TRIS buffer and filtering the solution through 450 nm filters. The variation of the S or PE concentrations (and, thus, the molar ratio between S or PE and POPC, denoted as S:POPC or PE:POPC, respectively) was achieved by successive additions of the stock S (or PE) solution in 5 mM TRIS buffer into to the POPC suspension. The nominal S:POPC (PE:POPC) molar ratios were in the range of S:POPC = 0–125 (PE:POPC = 0–4).

#### 3.1.6. Cultures of Microalgae

Cultures of microalgae *Dunaliella tertiolecta* (CCAP 19/22) and *Phaeodactylum tricornutum* (CCAP 1052/1A) were from the Culture Collection of Algae and Protozoa (CCAP) at SAMS (Oban, UK) and were a kind gift of the Microalgal Molecular Ecology and Biotechnology Unit, IT Sligo, Department of Environmental Science, Sligo, Ireland. They were grown in seawater (distilled water with added salt from Piran salt fields 22 g/L; autoclaved and filtered through 0.2-micron filters, 11107-47-ACN), enriched with Guillard’s (F/2) mixture of nutrients, minerals, and vitamins (Sigma Aldrich, USA, G0154, lot RNBG2437), diluted according to the manufacturer’s instructions (20 mL/L marine water) in a respirometer (Echo, Slovenia) at 20 °C with 20% illumination (approx. 250 μmol/m^2^s) with a 14 h/10 h (light/dark) lighting cycle and an airflow of 0.2 L/min.

#### 3.1.7. Preparation of Cells for the Study of Effects of Surfactants

For erythrocytes, a droplet of blood was created by a puncture of the finger of one of the authors from which 20 μL was suspended in 1 mL of PBS-citrate buffer (154 mM NaCl, 1.4 mM phosphate, 10.9 mM trisodium citrate, pH 7.4). The sample was centrifuged at 100× *g* for 3 min. The supernatant was removed, and the erythrocytes were resuspended at a final concentration of approximately 1 × 10^6^ cells/mL. For microalgae, cells were obtained from 50 mL of each of the cultures in the exponential phase of growth (day 20 after inoculation into the bottles). Cells were harvested from the cultures by centrifugation at 100× *g* (*Dunaliella tertiolecta*) or 300× *g* (*Phaeodactylum tricornutum*) for 10 min at 22 °C. Cells were resuspended in marine water at a final concentration of approximately 1 × 10^6^ cells/mL. A surfactant solution of SDS or TX100 was added to 1 mL aliquots. Samples were analyzed by flow cytometry after 14–20 h incubation in the dark. The tests were carried out in two parallels.

#### 3.1.8. Preparation of Extracellular Vesicles from Erythrocytes

Blood was donated by an author with no record of disease, after 12 h of fasting by using a G21 needle (Microlance, Becton Dickinson, Franklin Lakes, NJ, USA) and 4.5 mL evacuated tube with trisodium citrate (BD Vacutainers, 367714A, Becton Dickinson, Franklin Lakes, NJ, USA). Blood was centrifuged at 300× *g* and 18 °C for 10 min in the Centric 400/R (Domel, Slovenia) centrifuge to sediment erythrocytes. The plasma was discarded and the erythrocytes were washed three times with PBS–citrate (137 mM NaCl, 2.68 mM KCl, 10.14 mM Na_2_HPO_4_, 1.84 mM KH_2_PO_4_, 1.03 mM Na_3_C_6_H_5_O_7_, pH 7.2). Washed erythrocytes were stored in a buffer solution at 4 °C for 6 days. After gentle homogenization of the sample by turning the tube upside down several times, the sample was subjected to sequential centrifugation at 500× *g*, 2000× *g*, and 4000× *g* at 4 °C; each step occurred for 10 min in the centrifuge Centric 400/R (Domel, Železniki, Slovenia). The supernatant was subjected to centrifugation at 4 °C and 50,000× *g* for 70 min in an ultracentrifuge Beckman L8–70M with rotor SW55Ti (Thermo Fisher Scientific, Waltham, MA, USA). The pellet was suspended in 5 mL of PBS–citrate, and ultracentrifugation was repeated at the same conditions. For treatment with surfactants, the resuspended pellet was added to the respective solution. Samples were kept at 4 °C until further processing.

### 3.2. Methods

#### 3.2.1. Conductivity Measurements

Conductivity measurements were used to determine the CMC values of both ionic surfactants, CPC and SDS, in 5 mM TRIS buffer at 25 °C. Note that the CMC of nonionic surfactants (such as TX100) does not depend on the ionic strength of the solutions. The CMC value of TX100 in water was considered in this case [36]. The conductivity of CPC and SDS solutions was measured at 25 °C using an ISKRA conductivity meter and a conductivity cell with a cell constant of approximately 1 cm^−^^1^ (the exact value was determined by calibration with an aqueous KCl solution of known concentration). The titration technique was used to change the surfactant concentration. A solution of surfactant with a concentration well above the CMC was gradually added to 15 mL of the 5 mM TRIS buffer so that the final volume of the solution was 17 mL and the final surfactant concentration was well above the CMC. After each addition, the conductivity was measured.

#### 3.2.2. Isothermal Titration Calorimetry (ITC) Measurements

The complex heat effects associated with (de)micellization of surfactants (Ss) and interaction of S (or PE) with POPC liposomes were measured using Nano ITC (TA Instruments, New Castle, DE, USA) and VP–ITC (MicroCal Inc., San Diego, CA, USA) microcalorimeters. The initial experiments at 25 °C were conducted on Nano ITC. Due to relatively small enthalpy (Δ*H*) values and considerable noise, the experiments involving surfactants were further conducted on a VP–ITC at 15 °C where the heat associated with the (de)micellization is larger [25].

The titration cells with an effective cell volume of 0.95 mL (Nano ITC) and 1.39 mL (VP–ITC) were filled with POPC liposome suspension with the nominal POPC concentration (*c*_POPC_ = 0.66 mM) in the 5 mM TRIS buffer. The reference cell was filled with 5 mM TRIS buffer. Measurements on Nano ITC were performed at 25 °C by titrating a 10 mM (or 100 mM in case of SDS) S or PE stock solution in the same buffer in 4 µL aliquots with a 15-min interval by a 250 µL syringe while stirring at 250 rpm. The VP–ITC measurements were performed at 15 °C by titrating a 10 (or 100) mM S or PE stock solution in the same buffer in 3 µL aliquots with a 10-minute interval by a 300 µL syringe while stirring at 300 rpm.

The area under the peak after each injection of the S (or PE) stock solution, obtained by integrating the raw signal, was normalized by the amount of S (or PE) added and plotted against the S (or PE) concentration in the titration cell. Note that the concentration of the liposomes slightly decreases during the experiments due to the overflow/displacement design of the microcalorimeter cells [47]. The data were corrected for the blank heat effect accompanying the dilution of S or PE stock solutions. Heat effects accompanying dilution of the POPC liposome suspension were negligible.

#### 3.2.3. Light Scattering Measurements

Dynamic (DLS) and static light scattering (SLS) measurements were performed to determine the average hydrodynamic radius (*R*_h_) and the radius of gyration of (*R*_g_) of the POPC liposomes, respectively, and to measure the average intensity of light (*I*) scattered by the particles in the samples. Samples were analyzed with the 3D–DLS–SLS cross-correlation spectrometer from LS Instruments GmbH (Fribourg, Switzerland) using a 100 mW DPSS laser (Cobolt Flamenco, Cobolt AB, Stockholm, Sweden) with a wavelength λ_0_ = 660 nm at 25 °C. For processing the measured correlation functions (CFs), CONTIN analysis was used, which resulted in the intensity distribution of diffusion coefficients (*D*) of species in solution. The *R*_h_ values of liposomes were calculated from *D* using the Stokes–Einstein equation (*R*_h_ = *kT*6π*η*/D, where *k* is the Boltzmann constant, *T* is the absolute temperature, and *η* is the viscosity of the medium in which the particles diffuse), which assumes that the particles have a spherical shape. All calculations were carried out using the viscosity value of water at 25 °C. Details of this analysis can be found in the literature [48] and in SM accompanying this contribution. The value of *I* was interpreted as a measure of liposome concentration (in the case of preserved particle shape and size distribution) or topological change (in the case of a changed particle size distribution [48]).

Before the measurements, samples were carefully filtered through 450 nm Millex filters (Millipore, Burlington, MA, USA) directly into the measuring cuvette, which was inserted in a decalin bath and equilibrated at 25 °C for 15 min. In mixed S(PE)/POPC solutions, measurements were performed at a constant angle of 90°, whereas for the characterization of pure POPC liposomes (no added S or PE), they were carried out as a function of the angle of observation, i.e., in an angular range from 40° to 150° with a step of 10°. The constant intensity of the light scattered at 90° was used as a criterion that the solution was properly equilibrated. At each angle, five intensity CFs were recorded (60 s each) and averaged. Each curve was analyzed independently and compared to the average curve to ensure the accuracy of the mathematical solution.

Thermal stability analysis of POPC liposome suspensions, either pure or with the addition of S or PE at a nominal molar ratio S(PE):POPC = 1:1, was performed using the LitesizerTM 500 (Anton Paar GmbH, Graz, Austria). The 1 mL of a mixed S(PE):POPC suspension (*c*_POPC_ = 132 mM) was added to the cuvette. The sample was heated in 10 °C steps from 15 °C to 85 °C. After reaching the respective target temperature, the sample was equilibrated for another 5 min before 10 CFs of 20 s duration were recorded. The size distributions were determined from the mean CF using the Kalliope™ program (Anton Paar GmbH). The change in the *R*_h_ distribution and the change in the intensity of scattered light were followed.

#### 3.2.4. Flow Cytometry (FCM)

Cells (erythrocytes and microalgae) were analyzed by a MACS QUANT flow cytometer, Miltenyi, Bergisch-Gladbach, Germany). The following settings were used for blood sample analysis: FSC: 304 V; SSC: 454 V, trigger on SSC at 1.30 for cell analysis, and FSC: 458 V; SSC: 467 V, trigger on SSC at 1.80 for EP analysis. The following settings were used for microalgae analysis: FSC: 458 V, SSC: 467 V, B3: 216 V, R4: 400 V, trigger on SSC at 1.80. The measurements were taken in duplicate. The capture of plasma and algal samples is shown in Figures S6 and S7.

#### 3.2.5. Scanning Electron Microscopy (SEM)

Extracellular vesicles from washed erythrocytes were incubated overnight at 4 °C in a modified Karnovsky fixative (2.5% glutaraldehyde, 0.4% formaldehyde in PBS-citrate buffer). The fixative was removed by three steps of washing with distilled water (in each step, the sample was incubated for 10 min after changing the water), the samples were then incubated for 1 h in 2% OsO_4_, washed three times with distilled water (in each step the sample was incubated for 10 min after changing the distilled water), treated with a saturated solution of thiocarbohydrazide for 15 min, washed three times with distilled water (in each step, the samples were incubated for 10 min after changing the distilled water), and incubated again for 1 h in 2% OsO_4_. The osmium was then removed, and the samples were washed again three times and gradually dehydrated with a series of ethanol solutions in increasing concentrations (30–100%; in each step, the samples were incubated for 10 min after changing the solution, the step with absolute ethanol was repeated three times). Then, the samples were treated with hexamethyldisilazane in increasing concentrations (mixed with ethanol 30%, 50%, and 100%; in each step, the samples were incubated for 10 min after changing the solution), and finally air-dried. Algal samples were prepared according to an alternative procedure, where only 2% OsO_4_ was used for fixation, in which the samples were incubated for 2 h. The osmium was then removed, the samples were washed again three times, and gradually dehydrated with a series of ethanol solutions in increasing concentrations (30–100%; in each step, the sample was incubated for 10 min after changing the solution, the step with absolute ethanol was repeated three times). Then, the sample was treated with hexamethyldisilazane in increasing concentrations (mixed with ethanol 30%, 50%, and 100%; in each step, the sample was incubated for 10 min after changing the solution), and finally air-dried. The dried samples of erythrocyte extracellular vesicles and microalgae samples were sputtered with a mixture of gold and palladium and examined with a scanning electron microscope (JSM-6500F, JEOL Ltd., Tokyo, Japan).

## 4. Conclusions

In our combined light-scattering and microcalorimetry investigation of the effect of surfactants on the stability of POPC nanoliposomes, all three types of surfactants, cationic, anionic, and non-ionic, were included, unlike in conventional studies reported in the literature. We have shown that all employed surfactants had marked effects on the stability of simple POPC nanoliposomes. An important outcome of the study was that the uptake of surfactants by the liposomes was considerably accelerated if the concentration of the added surfactants exceeded their CMC values. The non-ionic TX100 exhibited the mildest effect, whereas the ionic surfactants in particular were very efficient in liposome degradation. On the other hand, anionic polyelectrolytes showed no such effect. The role of hydrophilic polymers, such as NaPSS and NaPMA in our study, was mainly to stabilize the liposomes via electrostatic interactions and adsorption of the PE chain on the liposome surface, which was consistent with rare studies in the literature including other compounds. Parallel microcalorimetry measurements of surfactant de-micellization and incorporation into the POPC membranes provided important insights into the mechanism of liposome degradation, a chief improvement in comparison with other studies. A central microcalorimetry result was that thermal response was governed by the following two factors: by the surfactant concentration (which needed to be above the CMC) and by the S:POPC molar ratio. Thus, our microcalorimetry (i.e., ITC) study provides important guidelines for the use of ITC for further research in the field of liposome–surfactant interactions.

In the second part of our study, we demonstrated that ionic (SDS) and non-ionic (TX100) surfactants caused micro-vesiculation of the cellular membrane, which is a much more complex assembly than simple POPC liposomes. This result supports the hypothesis that matter and information are transmitted at the cellular level in the form of nano-sized amphiphilic particles. Our results indicate that the essential parameter that determines the interaction of micelles with the membrane, either artificial or biological, is the match of the intrinsic curvature of the micelle and the local curvature of the membrane. We believe that our work contributes to the understanding of the mechanisms underlying delivery to cells and the knowledge in the field of biophysics of membranous nanostructures in general.

## Data Availability

Data are contained within the article and Supplementary Materials.

## Supplementary Materials

The following supporting information can be downloaded at https://www.mdpi.com/article/10.3390/molecules29194590/s1, Figure S1: CMC determination for ionic surfactants; Figure S2: structural characterization of liposomes; Figures S3–S5: Microcalorimetric measurements at 25 °C and 15 °C; Figures S6 and S7: Gating strategy in the analysis by FCM; Table S1: Rh, Rg and p values of pure POPC liposomes; Tables S2–S6: Rh and LS intensity values of POPC liposomes’ suspensions in the presence of surfactants and polyelectrolytes. Refs. [21,23,35,36,48,49,50,51,52] are cited in Supplementary Materials.

## References

[1] Welsh A.J., Goberdhan C.D., O′Driscoll L., Théry C., Witwer W.K. (2024). MISEV 2023: An Updated Guide to EV Research and Applications. J. Extracell. Vesicles.

[2] Gaurav I., Thakur A., Iyaswamy A., Wang X., Chen X., Yang Z. (2021). Factors Affecting Extracellular Vesicles Based Drug Delivery Systems. Molecules.

[3] Rajput A., Varshney A., Bajaj R., Pokharkar V. (2022). Exosomes as New Generation Vehicles for Drug Delivery: Biomedical Applications and Future Perspectives. Molecules.

[4] Sędzik M., Rakoczy K., Sleziak J., Kisiel M., Kraska K., Rubin J., Łuniewska W., Choromańska A. (2024). Comparative Analysis of Exosomes and Extracellular Microvesicles in Healing Pathways: Insights for Advancing Regenerative Therapies. Molecules.

[5] Pasarin D., Ghizdareanu A.-I., Enascuta C.E., Matei C.B., Bilbie C., Paraschiv-Palada L., Veres P.-A. (2023). Coating Materials to Increase the Stability of Liposomes. Polymers.

[6] Isomaa B., Hägerstrand H., Paatero G. (1987). Shape transformations induced by amphiphiles in erythrocytes. Biochim. Biophys. Acta..

[7] Isomaa B., Hägerstrand H. (1988). Effects of nonionic amphiphiles at sublytic concentrations on the erythrocyte membrane. Cell Biochem. Funct..

[8] Pandur Z., Penic S., Iglic A., Kralj-Iglic V., Stopar D., Drab M. (2023). Surfactin molecules with a cone-like structure promote the formation of membrane domains with negative spontaneous curvature and induce membrane invaginations. J. Coll. Int. Sci..

[9] Arrigler V., Kogej K., Majhenc J., Svetina S. (2005). Interaction of cetylpyridinium chloride with giant lipid vesicles. Langmuir.

[10] Jicsinszky L., Martina K., Cravotto G. (2021). Cyclodextrins in the antiviral therapy. J. Drug. Deliv. Sci. Technol..

[11] Fromm-Dornieden C., Rembe J.D., Schäfer N., Böhm J., Stuermer E.K. (2015). Cetylpyridinium chloride and miramistin as antiseptic substances in chronic wound management—prospects and limitations. J. Med. Microbiol..

[12] Alvarez D.M., Duarte L.F., Corrales N., Smith P.C., González P.A. (2020). Cetylpyridinium chloride blocks herpes simplex virus replication in gingival fibroblasts. Antivir. Res..

[13] Pérez-Errázuriz S., Velasco-Ortega E., Jiménez-Guerra A., Aguilera-Navarro E. (2021). Cetylpyridinium chloride as a tool against COVID-19. Int. J. Odontostomat..

[14] Parker W., Song P.S. (1992). Protein structures in SDS micelle-protein complexes. Biophys, J..

[15] Koley D., Bard A.J. (2010). Triton X-100 concentration effects on membrane permeability of a single HeLa cell by scanning electrochemical microscopy (SECM). Proc. Natl. Acad. Sci. USA.

[16] Borner M.M., Schneider E., Pirnia F., Sartor O., Trepel J.B., Myers C.E. (1994). The detergent Triton X-100 induces a death pattern in human carcinoma cell lines that resembles cytotoxic lymphocyte-induced apoptosis. FEBS Lett..

[17] Dayeh V.R., Chow S.L., Schirmer K., Lynn D.H., Bols N.C. (2004). Evaluating the toxicity of Triton X-100 to protozoan, fish, and mammalian cells using fluorescent dyes as indicators of cell viability. Ecotoxicol Env. Saf..

[18] Yasuhara K., Morigaki K. (2022). Creation of supramolecular biomembrane by the bottom-up self-assembly: Where material science meets biophysics. Biophys. Physicobiol..

[19] Yasuhara K., Arakida J., Ravula T., Ramadugu S., Sahoo B., Kikuchi J., Ramamoorthy A. (2017). Spontaneous lipid nanodisc fomation by amphiphilic polymethacrylate copolymers. J. Am. Chem. Soc..

[20] Coleman R., Holdsworth G. (1975). Effects of detergents on erythrocyte membranes: Different patterns of solubilization of the membrane proteins by dihydroxy and trihydroxy bile salts. Biochem. Soc. Trans..

[21] Škerjanc J., Kogej K., Vesnaver G. (1988). Polyelectrolyte-surfactant interactions, Enthalpy of binding of dodecyl- and cetylpyridinium cations to poly(styrenesulfonate) anion. J. Phys. Chem..

[22] Kogej K. (2010). Association and structure formation in oppositely charged polyelectrolyte-surfactant mixtures. Adv. Colloid Interface Sci..

[23] Božič D., Sitar S., Junkar I., Štukelj R., Pajnič M., Žagar E., Kralj-Iglič V., Kogej K. (2019). Viscosity of Plasma as a Key Factor in Assessment of Extracellular Vesicles by Light Scattering. Cell.

[24] Kogej K. (2016). Thermodynamic analysis of the conformational transition in aqueous solutions of isotactic and atactic poly(methacrylic acid) and the hydrophobic effect. Polymers.

[25] Medoš Ž., Plechkova N.V., Friesen S., Buchner R., Bešter-Rogač M. (2019). Insight into the hydration of cationic surfactants: A thermodynamic and dielectric study of functionalized quaternary ammonium chlorides. Langmuir.

[26] Kralj-Iglič V., Pocsfalvi G., Mesarec L., Šuštar V., Hägerstrand H., Iglič A. (2020). Minimizing isotropic and deviatoric membrane energy—An unifying formation mechanism of different cellular membrane nanovesicle types. PLoS ONE.

[27] Picciotto S., Barone M.E., Fierli D., Aranyos A., Adamo G., Božič D., Romancino D.P., Stanly C., Parkes R., Morsbach S. (2021). Isolation of extracellular vesicles from microalgae: Towards the production of sustainable and natural nanocarriers of bioactive compounds. Biomater. Sci..

[28] Adamo G., Fierli D., Romancino D.P., Picciotto S., Barone M.E., Aranyos A., Božič D., Morsbach S., Raccosta S., Stanly C. (2021). Nanoalgosomes: Introducing extracellular vesicles produced by microalgae. J. Extracell. Vesicles.

[29] Božič D., Hočevar M., Jeran M., Kisovec M., Bedina Zavec A., Romolo A., Škufca D., Podobnik M., Kogej K., Iglič A. (2022). Ultrastructure and stability of cellular nanoparticles isolated from *Phaeodactylum tricornutum* and *Dunaliella tertiolecta* conditioned media. Open Res Eur..

[30] Romolo A., Jan Z., Bedina Zavec A., Kisovec M., Arrigler V., Spasovski V., Podobnik M., Iglič A., Pocsfalvi G., Kogej K. (2022). Assessment of Small Cellular Particles from Four Different Natural Sources and Liposomes by Interferometric Light Microscopy. Int. J. Mol. Sci..

[31] Štibler U., Božič D., Hočevar M., Jeran M., Touzet N., Manno M., Pocsfalvi G., Bongiovanni A., Iglič A., Kralj-Iglič V. (2020). Toxicity of surfactants sodium dodecyl sulphate and TRITON X-100 to marine microorganisms. Proc. Socrat. Lect..

[32] Invally K., Ju L.K. (2017). Biolytic effect of rhamnolipid biosurfactant and dodecyl sulfate against Phagotrophic alga Ochromonas danica. J. Surfactants Deterg..

[33] Masakorala K., Turner A., Brown M.T. (2011). Toxicity of synthetic surfactants to the marine macroalga, Ulva lactuca. Water Air Soil Pollut..

[34] Cserháti T., Forgács E., Oros G. (2002). Biological activity and environmental impact of anionic surfactants. Environ Int..

[35] Motin M.A., Hafiz M.K.M.S., Reza N., Islam M.A., Yousuf M.A. (2012). Effect of Sodium Dodecyl Sulfate on Volumetric Properties of Methanol Ethanol n-Propanol and iso-Propanol at (298.15–323.15) K. Salam Dhaka. Univ. J. Sci..

[36] Tiller G., Mueller T., Dockter M., Struve W. (1984). Hydrogenation of Triton X-100 eliminates its fluorescence and ultraviolet light absorption while preserving its detergent properties. Anal. Biochem..

[37] Chen M., Zeng Z., Qu X., Tang Y., Long Q., Feng X. (2015). Biocompatible anionic polyelectrolyte for improved liposome based gene transfection. Int. J. Pharm..

[38] Tasi L.M., Liu D.Z., Chen W.Y. (2003). Microcalorimetric investigation of the interaction of polysorbate surfactants with unilamellar phosphatidylcholines liposomes. Coll. Surf. A Physicochem. Eng. Asp..

[39] Kawakami K., Nishihara Y., Hirano K. (2001). Effect of Hydrophilic Polymers on Physical Stability of Liposome Dispersions. J. Phys. Chem. B.

[40] Chern C.S., Chiu H.C., Yang Y.S. (2006). Interactions between nonionic Triton X surfactants and cholesterol-containing phosphatidylcholine liposomes. J. Coll. Int. Sci..

[41] Tesson B., Gennet M.J., Fernandez V., Degand S. (2009). Surface Chemical Composition of Diatoms. Chem. Bio. Chem..

[42] Tinaïg L.C., Carlos U., Lalia M., William H. (2017). New structural insights into the cell-wall polysaccharide of the diatom Phaeodactylum tricornutum. Algal Res..

[43] Chen H., Jiang J.-G. (2009). Osmotic responses of Dunaliella to the changes of salinity. J. Cell Physiol..

[44] Zetsche E.M., Meysman F.J.R. (2012). Dead or alive? Viability assessment of micro- and mesoplankton. J. Plankton Res..

[45] Wright D.A., Gensemer R.W., Mitchelmore C.L., Stubblefield W.A., van Genderen E., Dawson R., Orano-Dawson C.E., Bearr J.S., Mueller R.A., Cooper W.J. (2010). Shipboard trials of an ozone-based ballast water treatment system. Mar. Pollut. Bull..

[46] Darzynkiewicz Z., Li X., Gong J. (1994). Assays of cell viability: Discrimination of cells dying by apoptosis. Methods Cell Biol..

[47] Tellinghuisen J. (2007). Calibration in isothermal titration calorimetry: Heat and cell volume from heat of dilution of NaCl(aq). Anal. Biochem..

[48] Schärtl W. (2007). Light Scattering from Polymer Solutions and Nanoparticle Dispersions.

[49] Qazi M.J., Liefferink R.W., Schlegel S.J., Backus E.H.G., Bonn D., Shahidzadeh N. (2017). Influence of Surfactants on Sodium Chloride Crystallization in Confinement. Langmuir ACS J. Surf. Colloids.

[50] Brown W. (1993). Dynamic Light Scattering: The Method and Some Application.

[51] Hriberšek P., Kogej K. (2019). Tacticity and counterion modulated temperature response of weak polyelectrolytes: The case of poly(methacrylic acid) stereoisomers in aqueous solutions. Macromolecules.

[52] Sitar S., Aseyev V., Kogej K. (2014). Microgel-like aggregates of isotactic and atactic poly(methacrylic acid) chains in aqueous alkali chloride solutions as evidenced by ligh scattering. Soft Matter..

