# Supplementary material for: The Effect of Different Surfactants and Polyelectrolytes on Nano-Vesiculation of Artificial and Cellular Membranes
Source: Molecules. 2024 Sep 27;29(19):4590. doi: 10.3390/molecules29194590 (PMC11477677; doi:10.3390/molecules29194590)
Supplement: Supplementary file 1 [file molecules-29-04590-s001.zip › molecules-3210424-supplementary.pdf]

*Supporting material for the paper*

# The Effect of Different Surfactants and Polyelectrolytes on Nano-Vesiculation of Artificial and Cellular Membranes

**Urška Zagorc [1], Darja Božič [2], Vesna Arrigler [1], Žiga Medoš [1], Matej Hočevar [3], Anna Romolo [2], Veronika Kralj-Iglič [2,*] and Ksenija Kogej [1,*]**

[1] University of Ljubljana, Faculty of Chemistry and Chemical Technology, Chair for Physical Chemistry, SI-1000 Ljubljana, Slovenia

[2] University of Ljubljana, Faculty of Health Sciences, Laboratory of Clinical Biophysics, SI-1000 Ljubljana, Slovenia

[3] Institute of Metals and Technology, SI-1000 Ljubljana, Slovenia

* Correspondence: kraljiglic@gmail.com (V.K.-I.); ksenija.kogej@fkkt.uni-lj.si (K.K.)

## Determination of the critical micelle concentration (CMC) of ionic surfactants

The addition of salts (TRIS buffer components) to ionic surfactants (CPC and SDS) reduces the electrostatic repulsion between the charged functional groups in the headgroups of the molecules when they form micelles. This facilitates the formation of ionic surfactant micelles and decreases their CMC. In the case of the non-ionic surfactant Triton X-100, the effect of salt on the CMC is expected to be negligible [1]. Therefore, only the CMC values of CPC and SDS in 5 mM TRIS buffer were determined using conductivity measurements. Figure S1 shows the conductivity (in µS/cm) of the CPC and SDS solutions in 5 mM TRIS buffer as a function of surfactant concentration. The CMC was determined from the break in the curves (i.e., the change in slope below and above the CMC). The following values were obtained: CMC = 0.6 mM (600 µM) for CPC and 7.55 mM (7550 µM) for SDS, both in 5 mM Tris buffer at 25°C. These values are slightly lower than those in water at the same temperature (25°C)—CMC (CPC) = 0.63 mM [2] and CMC (SDS) = 8.2 mM [3]—which is expected given the low buffer concentration, i.e., the low ionic strength of the solution. The CMC value of Triton X-100 in water at 25°C is between 0.22 and 0.24 mM (220-240 µM), which is the lowest of all the surfactants used in this study [4] and was taken as being independent of ionic strength.

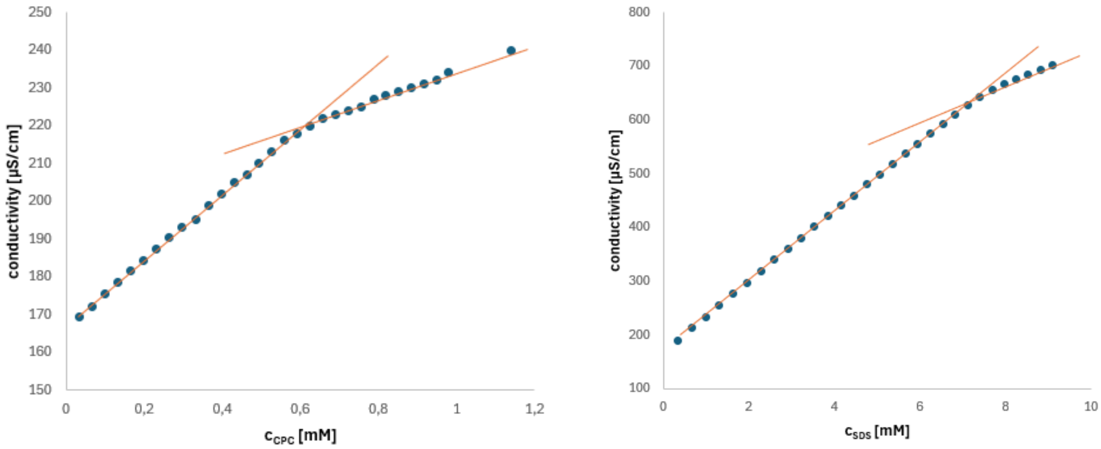


**Figure S1.** Dependence of conductivity in CPC (left panel) and SDS solution (right panel) on surfactant concentration in 5 mM Tris buffer with pH = 8 at 25 °C. The CMC was determined as the intersection of the orange lines.

**Characterization of pure POPC liposome suspensions**

Vesicle size and polydispersity are usually controlled by extrusion. However, the obtained size and polydispersity are very rarely checked after extrusion. We employed DLS and SLS to determine the size and shape of POPC, which was used for further measurements. An example of the measured correlation function $G_2(t)$ (*c.f.* Figure S2A) and the resulting $R_h$ distribution (*c.f.* Figure S2A, inset) determined by DLS at an angle of 90° are shown in Figure S2A (similar results were obtained at other angles). $G_2(t)$ and $R_h$ distribution indicate no sign of aggregation in the pure POPC suspensions in 5 mM TRIS buffer. For details on DLS analysis, see refs. [5,6]. The distribution is rather narrow (the polydispersity index PDI ≈ 0.1 – 0.17) with an average hydrodynamic radius $R_h$ of POPC at this angle being around 60 nm, i.e., a diameter $D_h$ around 120 nm. The measured vesicle size is thus expected, and is somewhat larger than the pore size of the membranes used for extrusion (100 nm).

An example of the dependency of $R_h$ and the relaxation rate Γ (= $Dq^2$) on the square of the scattering vector $q^2$ (where $q$ is related to the angle of observation θ and the refractive index of the medium $n_0$ by $q = 4\pi n_0/\lambda_0)sin(\theta/2)$) is shown in Figure S2B (solid blue circles). We see that $R_h$ slowly decreases with increasing $q^2$, whereas Γ shows an almost linear dependency on $q^2$ that passes through the center of the coordinates, a feature that is characteristic of the diffusive motion of particles. A slight positive curvature of the $\Gamma = f(q^2)$ dependency at higher $q$ (higher angles), together with the decreasing $R_h(q^2)$ dependency, is a consequence of the small degree of polydispersity of the vesicle population.

Along with the DLS part, the average LS intensity (the SLS part) was measured as a function of $q$ (or θ) to determine the radius of gyration ($R_g$) of POPC through the analysis of the form factor $P(q)$. $P(q)$ (= $I(q)/I(0)$) was calculated from the scattered light intensity $I(q)$, and was measured at a scattering vector $q$ (or angle θ) and $I(0)$, which was obtained by extrapolating $I(q^2)$ to $q = 0$. Examples of the $P(q)$ and the inverse of the LS intensity $(R-R_0)^{-1}$ (the difference $(R-R_0)$ is the LS intensity in absolute units, where $R$ is the Rayleigh ratio of the solution and $R_0$ is that of the solvent) as a function of $q^2$ are shown in Figure S2C. For details on the SLS analysis, see [5-9].

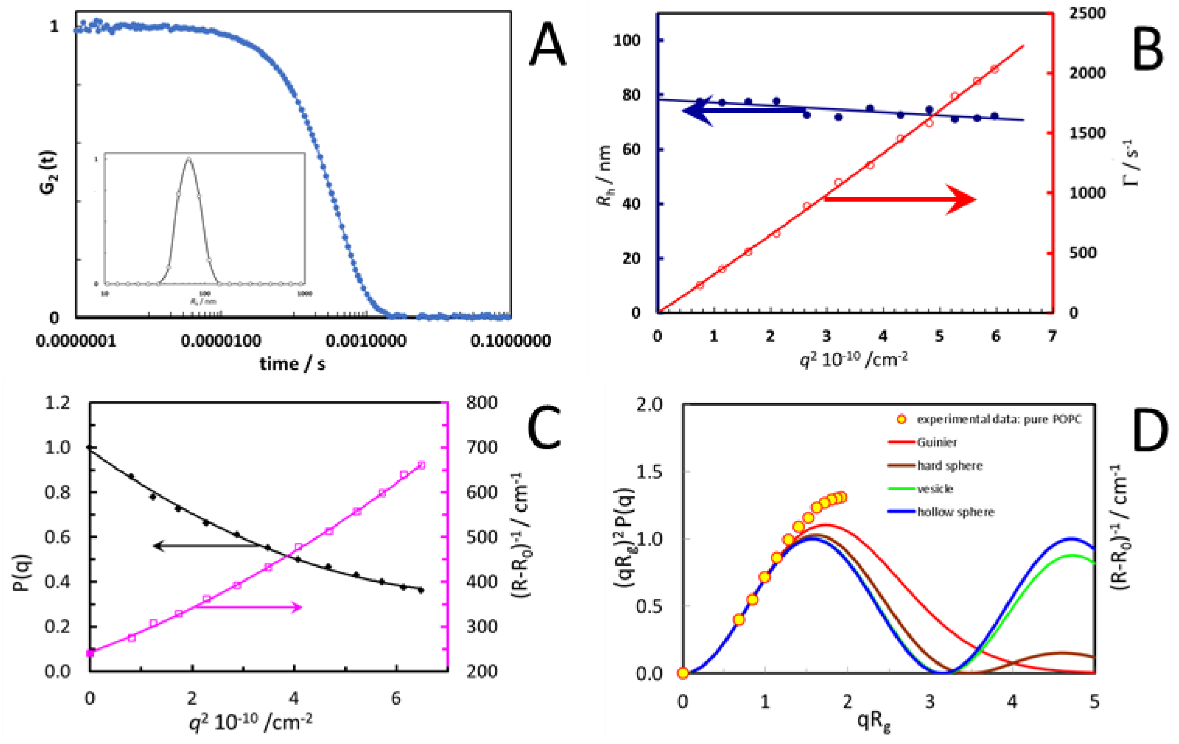


**Figure S2.** Dynamic and static light scattering analysis of POPC suspensions in 5mM Tris buffer. A) correlation function $G_2(t)$ and the intensity distribution of $R_h$ values (both DLS results; for $R_h$, see inset) of vesicle population in pure POPC suspension in 5 mM TRIS buffer at 25°C; B) dependence of $R_h$ and $\Gamma$ on the square of the scattering vector $q$; C) dependence of the form factor $P(q)$ and of the inverse of the LS intensity $(R\text{-}R_0)^{-1}$ on the square of the scattering vector $q^2$; D) Kratky plot, i.e., the dependence of $(qR_g)^2P(q)$ on $qR_g$ for four selected topologies and the experimental data for POPC (circles).

$R_g$ was obtained from $P(q)$ by applying an appropriate expression for $P(q)$ as a function of $q^2$. For this purpose, so-called Guinier scattering functions were used, which are valid for spherical particles [5,6]. It is convenient to present the SLS data in the form of a Kratky plot (dependence of $(qR_g)^2P(q)$ on $qR_g$). A comparison of the experimental data for the POPC suspension in 5 mM TRIS buffer with the calculated curves for some typical spherical particle topologies that are relevant for this case (hard sphere, vesicle, and hollow sphere) are also plotted in Figure S2D. Due to the relatively small size of $D_h$ = 120 nm and the employed wavelength of light ($\lambda_0$ = 660 nm), the experimental points are limited to $qR_g$ values below 2, where the differences between particle topologies are not yet very pronounced, but it is clearly seen that the experimental data display positive deviations from the calculated curves, e.g., for a monodisperse population (c.f. green line). Positive deviations are normally attributed to polydispersity [5,6], which is the case here.

The determined $R_h(0)$ (a value obtained by the extrapolation of $R_h = f(q^2)$ to $q = 0$; Figure S4B) and $R_g$ values for pure POPC vesicle suspensions in 5 mM TRIS buffer are collected in Table S1. For the case shown in Figure S2, the values are as follows: $R_h(0) = 78.5$ nm and $R_g = 76.4$ nm. From this, one obtains a shape parameter $\rho = R_g/R_h(0)$ equal to 0.97, which is expected for spherical particle topologies with a low internal density and mass concentrated on the rim (the theoretical ρ for a hollow spherical particle is $\rho = 1$ [5]). Other values are reported in Table S1.

**Table S1.** Hydrodynamic radius at $q = 0$ ($R_h(0)$), radius of gyration ($R_g$), and the shape parameter $\rho = R_g/R_h(0)$ of POPC in 5 mM TRIS buffer at pH = 8 and 25°C.

| $R_h(0)$ / nm | $R_g$ / nm | $\rho = R_g/R_h(0)$ |
|---|---|---|
| 78.5 | 78.4 | 1.00 |
| 78.5 | 75.6 | 0.96 |
| 75.3 | 75.5 | 1.00 |
| 69.7 | 68.4 | 0.98 |

**Effect of surfactants and polyelectrolytes on liposome size and stability**

The results of DLS measurements (the average $R_h$ values of the POPC liposome population and the total intensity of scattered light, $I_{tot}$, from POPC suspensions at an angle of 90°) are collected in Tables S2-S8 for all studied systems.

**Table S2A.** Hydrodynamic radius ($R_h$) and total LS intensity ($I_{tot}$) for POPC liposome suspensions in 5 mM Tris buffer with pH = 8 at 25°C in the presence of CPC. $R_h/R_h(0)$ is the ratio between the hydrodynamic radius of POPC with ($R_h$) and without ($R_h(0)$) added CPC, and $I_{tot}/I_{tot}(0)$ is the corresponding ratio of the total LS intensity. The nominal concentration of POPC is $c_{POPC}$ = 132 μM. The results are shown for the first and second series of measurements.

| $c_{CPC}$ [μM] | Series 1 | | | | Series 2 | | | |
|---|---|---|---|---|---|---|---|---|
| | $R_h$ / nm | $\frac{R_h}{R_h(0)}$ | $I_{tot}$ / kHz/mW | $\frac{I_{tot}}{I_{tot}(0)}$ | $R_h$ / nm | $\frac{R_h}{R_h(0)}$ | $I_{tot}$ / kHz/mW | $\frac{I_{tot}}{I_{tot}(0)}$ |
| 0 | 61 | 1 | 112 | 1 | 64 | 1 | 66 | 1 |
| 33* | 66 | 1.08 | 94 | 0.84 | | | | |
| 66 | 60 | 0.98 | 94 | 0.84 | | | | |
| 99* | 71 | 1.16 | 102 | 0.91 | 52 | 0.81 | 75 | 1.14 |
| 132* | 67 | 1.10 | 99 | 0.88 | | | | |
| 198 | 63 | 1.03 | 102 | 0.91 | | | | |
| 264 | 65 | 1.06 | 103 | 0.92 | | | | |
| 330 | 65 | 1.06 | 92 | 0.82 | 52 | 0.81 | 67 | 1.02 |
| 396 | 61 | 1 | 75 | 0.67 | | | | |
| 660 | | | | | 72 | 1.12 | 76 | |
| 792 | | | | | /** | /** | /** | |

*Smaller particles also present.

**Determination of $R_h$ was not possible.

**Table S2B.** Hydrodynamic radius ($R_h$) and total LS intensity ($I_{tot}$) for POPC liposome suspensions in 5 mM Tris buffer with pH = 8 at 25°, in the presence of CPC. $R_h/R_h(0)$ is the ratio between the hydrodynamic radius of POPC with ($R_h$) and without ($R_h(0)$) added CPC, and $I_{tot}/I_{tot}(0)$ is the corresponding ratio of the total LS intensity. The nominal concentration of POPC is $c_{POPC}$ = 132 μM. The results are shown for the third series of measurements.

| $c_{CPC}$ / μM | Series 3 | | | |
|---|---|---|---|---|
| | $R_h$ / nm | $\frac{R_h}{R_h(0)}$ | $I_{tot}$ / kHz/mW | $\frac{I_{tot}}{I_{tot}(0)}$ |
| 0 | 62.6 | 1 | 80 | 1 |
| 124 | 62.2 | 0.99 | 68 | 0.85 |
| 244 | 64.3 | 1.03 | 73 | 0.91 |
| 362 | 67.1 | 1.07 | 73 | 0.91 |
| 477 | 74.4 | 1.19 | 90 | 1.13 |
| 589* | 68.0 | 1.09 | 80 | 0.99 |
| 699* | 63.2 | 1.01 | 52 | 0.65 |
| 911* | 73.6 | 1.18 | 38 | 0.48 |
| 1113** | 82.5 | 1.32 | 34 | 0.42 |
| 1307** | 174 | 2.79 | 23.6 | 0.29 |

*Larger particles also present; increasingly polydisperse.

**Very polydisperse; $R_h$ given for the population with the largest contribution to $I_{tot}$.

**Table S2C.** Hydrodynamic radius ($R_h$) and total LS intensity ($I_{tot}$) for POPC liposome suspension in 5 mM Tris buffer with pH = 8 at 25°C immediately after CPC addition and after 24 hours. $R_h/R_h(0)$ is the ratio between the hydrodynamic radius of POPC with ($R_h$) and without ($R_h(0)$) added CPC, and $I_{tot}/I_{tot}(0)$ is the corresponding ratio of the LS intensity. The nominal concentration of POPC is $c_{POPC}$ = 132 μM. The results are shown for the second series of measurements.

| **Time / h** | $c_{CPC}$ **/ μM** | $R_h$ **/ nm** | $\frac{R_h}{R_h(0)}$ | $I_{tot}$ **/ kHz/mW** | $\frac{I_{tot}}{I_{tot}(0)}$ |
|---|---|---|---|---|---|
| 0 | 0 | 61 | 1.00 | 112 | 1 |
| 0 | 396 | 64 | 1 | 151 | 1 |
| 24 | 396 | 61* | 1 | 75 | 0.67 |
| 24 | 528 | 101* | 1.66 | 26 | 0.23 |

* Smaller particles also present.

**Table S2D.** Hydrodynamic radius ($R_h$) and total LS intensity ($I_{tot}$) for POPC liposome suspensions in 5 mM Tris buffer with pH = 8 at 25°C immediately after CPC addition and after 24 hours. $R_h/R_h(0)$ is the ratio between the hydrodynamic radius of POPC with ($R_h$) and without ($R_h(0)$) added CPC, and $I_{tot}/I_{tot}(0)$ is the corresponding ratio of the LS intensity. The nominal concentration of POPC is $c_{POPC}$ = 132 μM. The results are given for the third series of measurements.

| **Time / h** | $c_{CPC}$ **/ μM** | $R_h$ **/ nm** | $\frac{R_h}{R_h(0)}$ | $I_{tot}$ **/ kHz/mW** | $\frac{I_{tot}}{I_{tot}(0)}$ |
|---|---|---|---|---|---|
| 0 | 0 | 64 | 1.00 | 151 | 1.00 |
| 0 | 362 | 66 | 1.04 | 130 | 0.86 |
| 24 | 362 | 74 | 1.16 | 49 | 0.32 |
| 24 | 477 | 60 | 0.94 | 31 | 0.21 |

**Table S3A.** Hydrodynamic radii ($R_h$) of POPC liposomes and the total LS intensity ($I_{tot}$) in a suspension of 5 mM Tris buffer at pH = 8 in the presence of SDS. $R_h/R_h(0)$ is the ratio between the hydrodynamic radius of POPC with ($R_h$) and without ($R_h(0)$) added SDS, and $I_{tot}/I_{tot}(0)$ is the corresponding ratio of the total LS intensity. The nominal concentration of POPC is $c_{POPC}$ = 132 μM. The results are given for the first and second series of measurements.

| $c_{SDS}$ [μM] | Series 1 | | | | Series 2 | | | |
|---|---|---|---|---|---|---|---|---|
| | $R_h$ [nm] | $\frac{R_h}{R_{h,0}}$ | $I_{tot}$ [kHz/mW] | $\frac{I_{tot}}{I_{tot,0}}$ | $R_h$ [nm] | $\frac{R_h}{R_{h,0}}$ | $I_{tot}$ [kHz/mW] | $\frac{I_{tot}}{I_{tot,0}}$ |
| 0 | 61 | 1.00 | 112 | 1.00 | 64 | 1.00 | 66 | 1.00 |
| 66 | 70 | 1.15 | 109 | 0.97 | 67 | 1.05 | 63 | 0.95 |
| 132* | 86 | 1.41 | 107 | 0.96 | | | | |
| 264* | 73 | 1.20 | 106 | 0.95 | | | | |
| 396* | 68 | 1.11 | 105 | 0.94 | 67 | 1.05 | 72 | 1.09 |
| 528 | 68 | 1.11 | 106 | 0.95 | | | | |
| 660 | 74 | 1.21 | 106 | 0.95 | 57 | 0.89 | 75 | 1.14 |
| 990 | 67 | 1.10 | 106 | 0.95 | | | | |
| 1320 | 68 | 1.11 | 105 | 0.94 | 59 | 0.92 | 73 | 1.11 |
| 2640 | | | | | 72 | 1.13 | 90 | |
| 5280 | | | | | 53 | 0.83 | 36 | 0.55 |
| 7920* | | | | | 103 | 1.61 | 60 | 0.91 |
| 9240* | | | | | 70 | 1.09 | 49 | 0.74 |

*Larger particles also present; increasingly polydisperse.

**Very polydisperse; $R_h$ given for the population with the largest contribution to $I_{tot}$.

**Table S3B.** Hydrodynamic radii ($R_h$) of POPC liposomes and the total LS intensity ($I_{tot}$) in a suspension of 5 mM Tris buffer at pH = 8 in the presence of SDS. $R_h/R_h(0)$ is the ratio between the hydrodynamic radius of POPC with ($R_h$) and without ($R_h(0)$) added SDS, and $I_{tot}/I_{tot}(0)$ is the corresponding ratio of the total LS intensity. The nominal concentration of POPC is $c_{POPC}$ = 132 μM. The results are given for the third series of measurements.

| $c_{SDS}$ [μM] | Series 3 | | | |
|---|---|---|---|---|
| | $R_h$ [nm] | $\frac{R_h}{R_{h,0}}$ | $I_{tot}$ [kHz/mW] | $\frac{I_{tot}}{I_{tot,0}}$ |
| 0 | 71 | 1.00 | 117 | 1.00 |
| 125 | 67 | 0.94 | 108 | 0.92 |
| 622 | 68 | 0.96 | 126 | 1.08 |
| 1237 | 71 | 1.00 | 132 | 1.13 |
| 2444 | 68 | 0.96 | 137 | 1.17 |
| 4771 | 70 | 0.98 | 131 | 1.12 |
| 7422 | 73 | 1.03 | 117 | 1.00 |
| 8589 | 72 | 1.01 | 86 | 0.74 |
| 9624 | 67 | 0.95 | 54 | 0.46 |
| 11625 | 65 | 0.91 | 32 | 0.27 |
| 13541* | 80 | 1.13 | 31 | 0.26 |

*Larger particles also present.

**Table S3C.** Hydrodynamic radius ($R_h$) of POPC liposomes and total LS intensity ($I_{tot}$) as a function of time and concentration of added SDS in a POPC suspension with $c_{POPC}$ = 132 μM. $R_h/R_h(0)$ is the ratio between the hydrodynamic radius of POPC with ($R_h$) and without ($R_h(0)$) added CPC, and $I_{tot}/I_{tot}(0)$ is the corresponding ratio of the total LS intensity. The results are given for the third series of measurements.

| Time / h | $c_{SDS}$ [μM] | $R_h$ / nm | $\frac{R_h}{R_{h,0}}$ | $I_{tot}$ [kHz/mW] | $\frac{I_{tot}}{I_{tot,0}}$ |
|---|---|---|---|---|---|
| 0 | 0 | 64 | 1.00 | 151 | 1 |
| 0 | 4771 | 68 | 1.07 | 114 | 0.76 |
| 24 | 4771 | 70 | 1.09 | 55 | 0.37 |
| 24 | 7422 | /* | /* | 1 | 0.01 |

*$R_h$ could not be determined.

**Table S4A.** Hydrodynamic radius ($R_h$) of POPC liposomes and total LS intensity ($I_{tot}$) as a function of time and concentration of added TX100 in a POPC suspension with $c_{POPC}$ = 132 μM. $R_h/R_h(0)$ is the ratio between the hydrodynamic radius of POPC with ($R_h$) and without ($R_h(0)$) added CPC, and $I_{tot}/I_{tot}(0)$ is the corresponding ratio of the total LS intensity.

| $c_{TX100}$ [μM] | $R_h$ [nm] | $\frac{R_h}{R_{h,0}}$ | $I_{tot}$ [kHz/mW] | $\frac{I_{tot}}{I_{tot,0}}$ |
|---|---|---|---|---|
| 0 | 61 | 1.00 | 112 | 1.00 |
| 33* | 76 | 1.25 | 110 | 0.98 |
| 66* | 83 | 1.36 | 118 | 1.05 |
| 99 | 77 | 1.26 | 124 | 1.11 |
| 132 | 77 | 1.26 | 129 | 1.15 |
| 198 | 75 | 1.23 | 129 | 1.15 |
| 264* | 83 | 1.36 | 89 | 0.79 |
| 330* | 73 | 1.20 | 67 | 0.60 |
| 396 | 74 | 1.21 | 53 | 0.47 |

*Smaller particles also present.

**Table S4B.** Hydrodynamic radius ($R_h$) of POPC liposomes and total LS intensity ($I_{tot}$) as a function of time and concentration of added TX100 in a POPC suspension with $c_{POPC}$ = 132 μM. $R_h/R_h(0)$ is the ratio between the hydrodynamic radius of POPC with ($R_h$) and without ($R_h(0)$) added TX100, and $I_{tot}/I_{tot}(0)$ is the corresponding ratio of the total LS intensity.

| Time / h | $c_{TX100}$ [μM] | $R_h$ [nm] | $\frac{R_h}{R_{h,0}}$ | $I_{tot}$ [kHz/mW] | $\frac{I_{tot}}{I_{tot,0}}$ |
|---|---|---|---|---|---|
| 0 | 0 | 68 | 1.00 | 147 | 1.00 |
| 0 | 136 | 77 | 1.14 | 175 | 1.19 |
| 24 | 136 | 71 | 1.05 | 126 | 0.86 |
| 24* | 196 | 71 | 1.05 | 106 | 0.72 |

*Larger particles also present.

**Table S5.** Hydrodynamic radius ($R_h$) of POPC liposomes and total LS intensity ($I_{tot}$) in a POPC suspension with $c_{POPC}$ = 132 μM in 5 mM Tris buffer with pH = 8 with and without added polyelectrolytes (PE: NaPSS or NaPMA). $R_h/R_h(0)$ is the ratio between the hydrodynamic radius of POPC with ($R_h$) and without ($R_h(0)$) added TX100, and $I_{tot}/I_{tot}(0)$ is the corresponding ratio of the total LS intensity.

| $c_{PE}$ [μM] | NaPSS | | | | | NaPMA | | | |
|---|---|---|---|---|---|---|---|---|---|
| | $R_{h,1}$ [nm] | $R_{h,2}$ [nm] | $\frac{R_{h,}}{R_{h,0}}$ | $I_{tot}$ [kHz/mW] | $\frac{I_{tot}}{I_{tot,0}}$ | $R_h$ [nm] | $\frac{R_h}{R_{h,0}}$ | $I_{tot}$ [kHz/mW] | $\frac{I_{tot}}{I_{tot,0}}$ |
| 0 | | 61 | 1.00 | 112 | 1.00 | 61 | 1.00 | 112 | 1.00 |
| 66 | | 68 | 1.11 | 107 | 0.96 | 84 | 1.38 | 119 | 1.06 |
| 132 | | 72 | 1.18 | 107 | 0.96 | 67 | 1.10 | 119 | 1.06 |
| 198 | | 66 | 1.08 | 103 | 0.92 | 64 | 1.05 | 119 | 1.06 |
| 264 | 9 | 70 | 1.15 | 106 | 0.95 | 79 | 1.29 | 121 | 1.08 |
| 396 | 23 | 77 | 1.26 | 118 | 1.05 | 69 | 1.13 | 121 | 1.08 |
| 528 | 30 | 83 | 1.36 | 119 | 1.06 | 61 | 1.00 | 123 | 1.10 |

**Table S6.** Hydrodynamic radii ($R_h$) of POPC liposomes as a function of temperature ($T$) in POPC liposome suspensions with added surfactants (S: CPC, SDS, and TX100) and polyelectrolytes (PE:

NaPSS and NaPMA). The nominal S(PE)/POPC molar ratio is 1:1, and $c_{POPC}$ = 132 μM. The second column gives $R_h$ for pure POPC liposomes as a function of $T$.

| | $R_h$ [nm] | | | | | |
|---|---|---|---|---|---|---|
| **$T$ / °C** | **POPC** | **CPC** | **TX100** | **SDS** | **NaPSS** | **NaPMA** |
| 15 | 74 | 71 | 81 | 69 | 80 | 68 |
| 25 | 76 | 72 | 83 | 70 | 79 | 70 |
| 35 | 77 | 72 | 85 | 70 | 79 | 71 |
| 45 | 79 | 74 | 86 | 72 | 77 | 72 |
| 55 | 76 | 72 | 86 | 74 | 81 | 71 |
| 65 | 79 | 73 | 86 | 73 | 79 | 73 |
| 75 | 78 | 73 | 85 | 73 | 79 | 73 |
| 85 | 76 | 74 | 85 | 73 | 78 | 74 |

## Calorimetry

The obtained enthalpograms for experiments involving stock solutions of S titrated into the suspension of POPC liposomes are shown in Figures S3-S5 for two temperatures, namely 25°C (panel A) and 15°C (panel B). Each Figure contains two experiments—the de-micellization of the surfactant in 5 mM Tris buffer (open symbols) and the same surfactant stock solution titrated into the 0.66 mM POPC vesicle suspension.

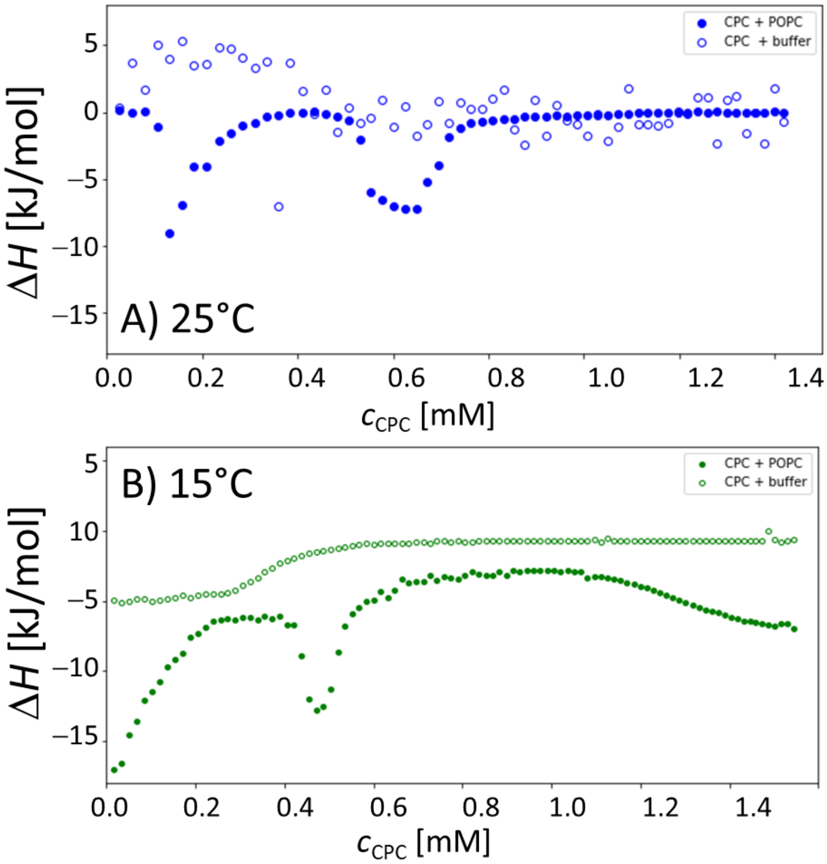


**Figure S3.** Enthalpograms obtained by titrating a 10 mM CPC into a 0.66 mM POPC suspension (filled circles) and into buffer (5 mM Tris; empty circles) on two microcalorimeters. A) Nano ITC: 25 °C; B) VP-ITC: 15 °C.

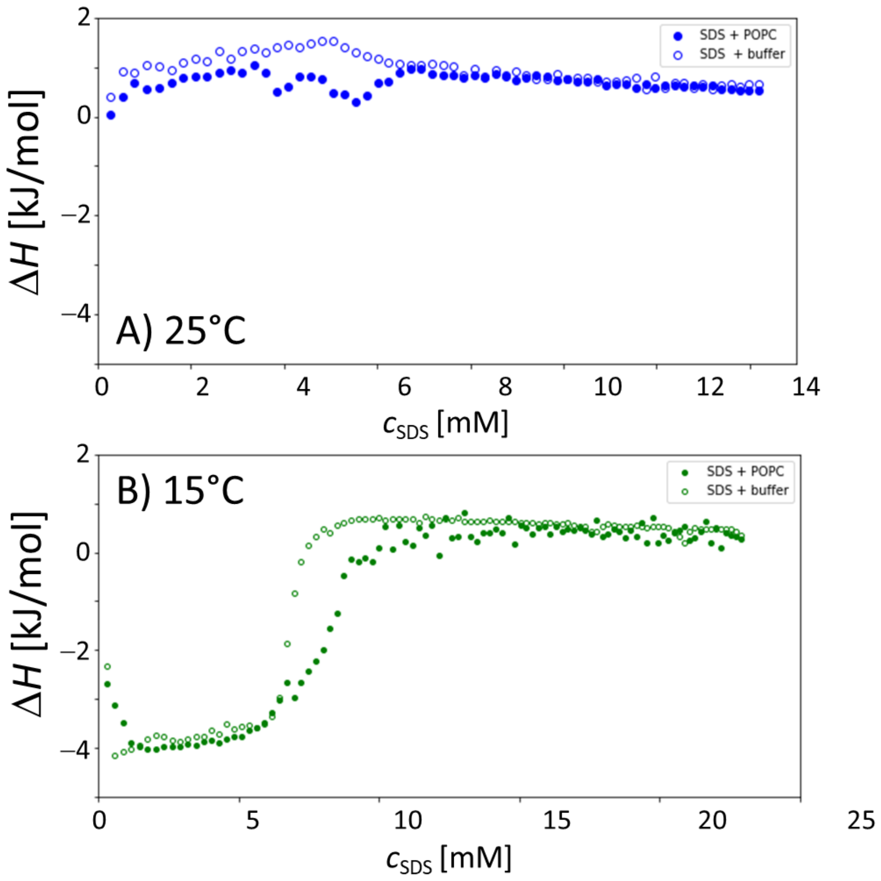


**Figure S4.** Enthalpograms obtained by titrating a 100 mM SDS into a 0.66 mM POPC suspension (filled circles) and into buffer (5 mM Tris; empty circles) on two microcalorimeters. A) Nano ITC: 25 °C; B) VP-ITC: 15 °C.

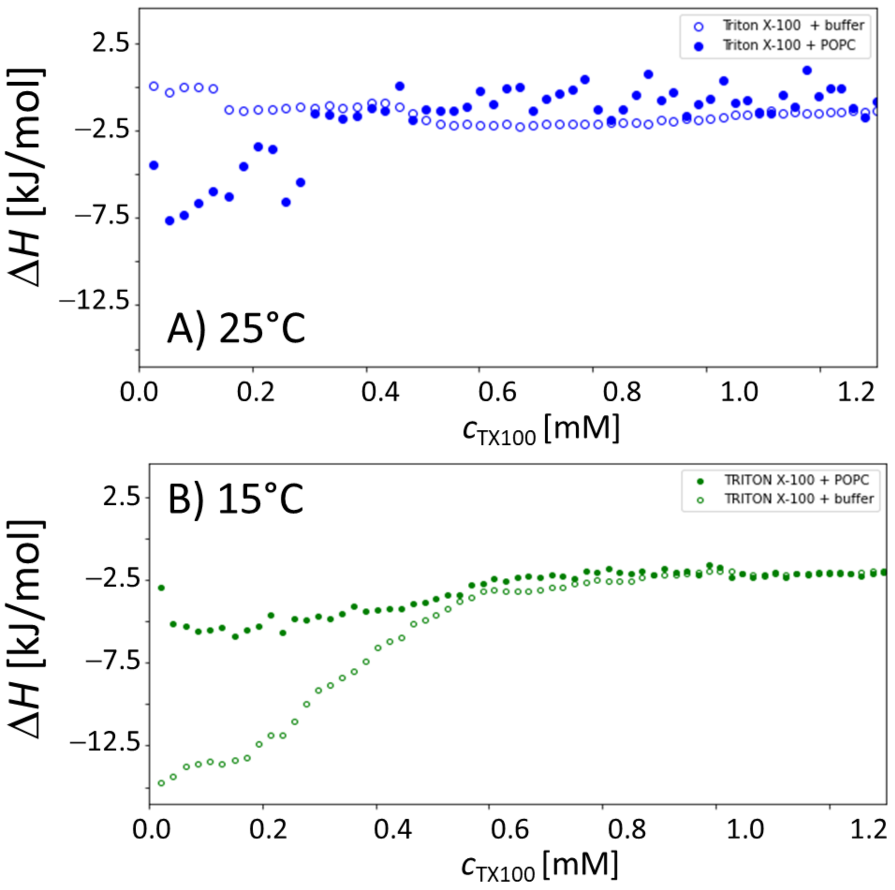


**Figure S5.** Enthalpograms obtained by titrating a 10 mM TX100 into a 0.66 mM POPC suspension (filled circles) and into buffer (5 mM Tris; empty circles) on two microcalorimeters. A) Nano ITC: 25 °C; B) VP-ITC: 15 °C.

**Flow cytometry (FCM) of blood cells and microalgae *Phaeodactylum tricornutum* and *Dunaliella tertiolecta***

The gating strategy in the analysis of FCM measurements of samples from erythrocyte-containing samples is shown in Figure S6. A) scatter diagram obtained with settings for cells; B) scatter diagram obtained with settings for larger EPs. We distinguished five particle populations. P1: mainly erythrocytes, but this region also contains leukocytes; P2 and P2* (in settings for larger and smaller particles, respectively): mainly platelets; Pa* + Pb*: large EPs; P3: weakly side-scattering particles.

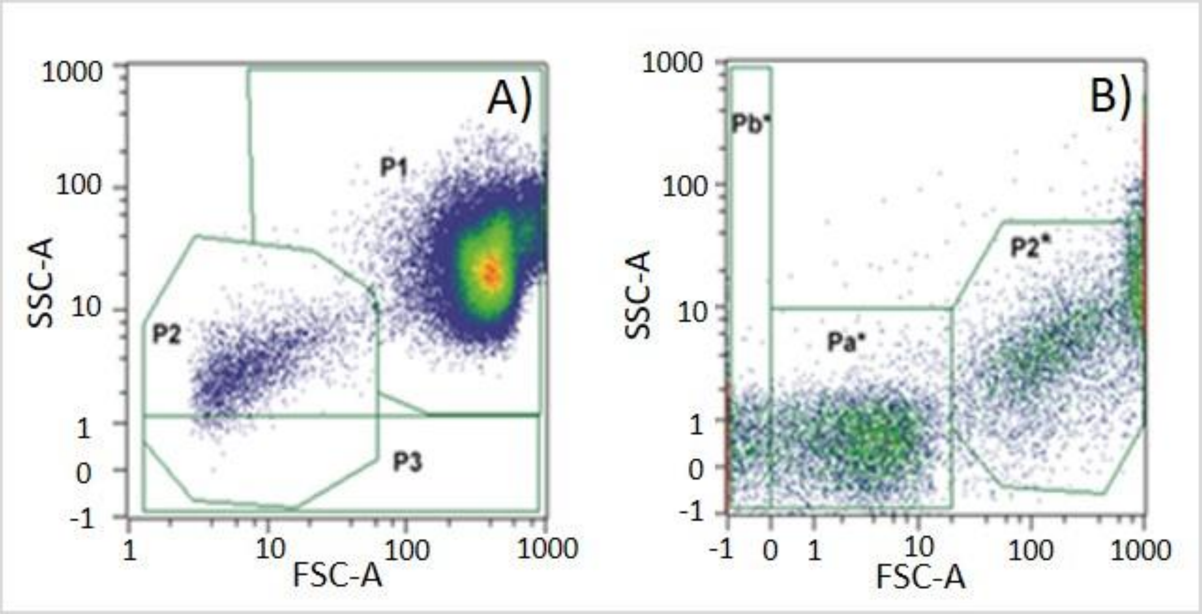


**Figure S6.** Gating strategy in the analysis of FCM measurements of blood. A) settings focused on cells; B) settings focused on the detection of larger EPs. Results pertaining to the measurements obtained in settings for the detection of larger EPs are indicated by an asterisk. P1: mainly erythrocytes and some leukocytes; P2, P2*: mainly platelets; Pa*, Pb*: large EPs; P3: weakly side-scattering particles. The color scale from blue to red corresponds to the increasing density of events.

The gating strategy in the analysis of FCM measurements of samples from microalgae cultures is shown in Figure S7. A), B): *Phaeodactylum tricornutum*; C), D): *Dunaliella tertiolecta*. Algal cells were identified based on chlorophyll autofluorescence in PI / PE-Cy5.5-A and APC-Cy7A channels (Panel A—*Pt*1, and Panel C—*Dt*1). EPs were characterized based on the anterior signal (FSC) and lateral (SSC) scattering (Panel B—*Pt*2, and Panel D—*Dt*2).

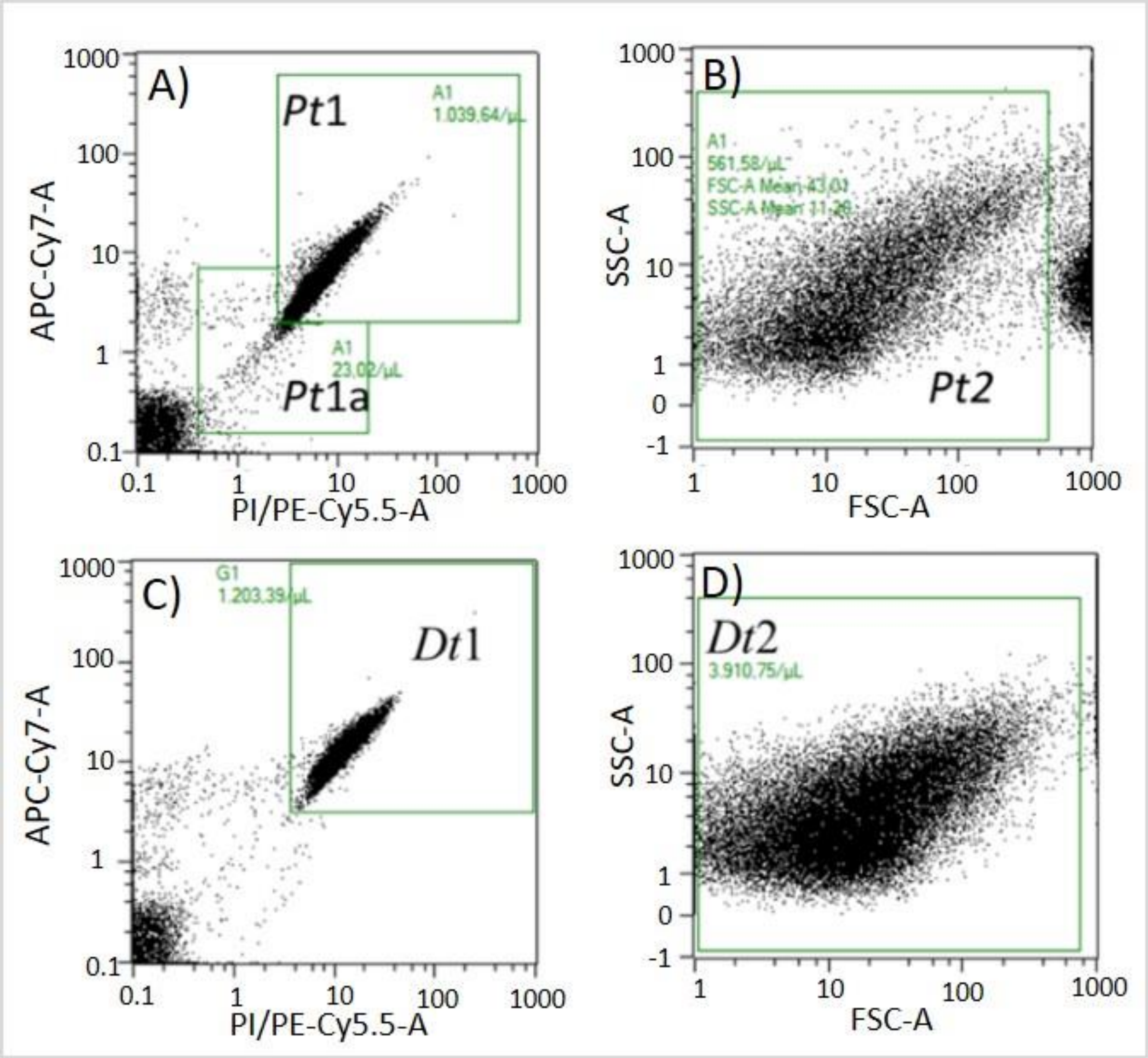


**Figure S7.** Gating strategy in the analysis of FCM measurements of samples from microalgae cultures. A), B): *Phaeodactylum tricornutum*; C), D): *Dunaliella tertiolecta*. Microalgal cells were identified based on chlorophyll autofluorescence in PI / PE-Cy5.5-A and APC-Cy7A channels (Panel A)—*Pt1*, and Panel C—*Dt1*). EPs were characterized based on the anterior signal (FSC) and lateral (SSC) scattering (Panel B—*Pt2*, and Panel D—*Dt2*).

**References**


[1] Qazi, M.J.; Liefferink, R.W.; Schlegel, S.J.; Backus, E.H.G.; Bonn, D.; Shahidzadeh, N. Influence of Surfactants on Sodium Chloride Crystallization in Confinement. Langmuir : the ACS journal of surfaces and colloids. **2017**, 33, 4260–4268.

[2] Skerjanc, J.; Kogej, K.; Vesnaver, G. Polyelectrolyte-Surfactant Interactions. Enthalpy of Binding of Dodecyl- and Cetylpyridinium Cations to Poly (Styrenesulfonate) Anion. Journal of physical chemistry. **1988**,92, 6382–6385.

[3] Motin, M.A.; MIA, M.; Reza, K.S.; Islam A.; Yousuf, M.; Salam, M. Effect of Sodium Dodecyl Sulfate on Volumetric Properties of Methanol Ethanol n-Propanol and iso-Propanol at (298.15 - 323.15) K. Dhaka University Journal of Science. **2012**.

[4] Tiller, G.E.; Mueller, T.J.; Dockter, M.E.; Struve, W.G. Hydrogenation of Triton X-100 Eliminates Its Fluorescence and Ultraviolet Light Absorption While Preserving Its Detergent Properties. Anal Biochem. **1984**,141, 262–266.

[5] Schärtl, W. Light Scattering from Polymer Solutions and Nanoparticle Dispersions. Springer Verlag: Berlin, Heidelberg, **2007**.

[6] Brown, W. Dynamic Light Scattering: The Method and Some Application. Clarendon Press: Oxford, **1993**.

[7] Hriberšek, P,; Kogej, K. Tacticity and counterion modulated temperature response of weak polyelectrolytes : the case of poly(methacrylic acid) stereoisomers in aqueous solutions. Macromolecules. **2019**, 52, 7028-7041.

[8] Sitar, S.; Aseyev, V.; Kogej. K. Microgel-like aggregates of isotactic and atactic poly(methacrylic acid) chains in aqueous alkali chloride solutions as evidenced by ligh scattering. Soft Matter. 2014, 10, 7712–7722.

[9] Božič, D.; Sitar, S.; Junkar, I.; Štukelj, R.; Pajnič, M.; Žagar, E.; Kralj-Iglič, V.; Kogej, K. Viscosity of Plasma as a Key Factor in Assessment of Extracellular Vesicles by Light Scattering. Cells. **2019**, 8, 1046.
